# Molecular recognition of ubiquitin and Lys63-linked diubiquitin by STAM2 UIM-SH3 dual domain: the effect of its linker length and flexibility

**DOI:** 10.1038/s41598-019-51182-0

**Published:** 2019-10-10

**Authors:** Minh-Ha Nguyen, Marie Martin, Henry Kim, Frank Gabel, Olivier Walker, Maggy Hologne

**Affiliations:** 1 Institut des Sciences Analytiques (ISA), Univ Lyon, CNRS, UMR5280, Université Claude Bernard Lyon 1, Lyon, France; 2 Université Grenoble Alpes, CEA, CNRS, IBS, 38042 Grenoble, France

**Keywords:** SAXS, Solution-state NMR

## Abstract

Multidomain proteins represent a broad spectrum of the protein landscape and are involved in various interactions. They could be considered as modular building blocks assembled in distinct fashion and connected by linkers of varying lengths and sequences. Due to their intrinsic flexibility, these linkers provide proteins a subtle way to modulate interactions and explore a wide range of conformational space. In the present study, we are seeking to understand the effect of the flexibility and dynamics of the linker involved in the STAM2 UIM-SH3 dual domain protein with respect to molecular recognition. We have engineered several constructs of UIM-SH3 with different length linkers or domain deletion. By means of SAXS and NMR experiments, we have shown that the modification of the linker modifies the flexibility and the dynamics of UIM-SH3. Indeed, the global tumbling of both the UIM and SH3 domain is different but not independent from each other while the length of the linker has an impact on the ps-ns time scale dynamics of the respective domains. Finally, the modification of the flexibility and dynamics of the linker has a drastic effect on the interaction of UIM-SH3 with Lys63-linked diubiquitin with a roughly eight-time weaker dissociation constant.

## Introduction

A wide ensemble of protein-protein interaction networks mediate communication and information exchange between cells^1^. Alteration or interruption in these networks usually leads to severe damage or disease^2,3^ and has led to a tremendous exploration of protein-protein inhibition^4,5^. These interactions are mainly mediated by protein domains, which could be considered as modular building blocks, assembled in different fashions. They decode specific signals emerging from post-translational modifications involved in receptor signaling, endocytosis or DNA damage for instance^6^. Like words and grammar that rule a language, protein domains may assemble differently and adopt different architectures^7,8^ to form multidomain proteins (MDPs) that represent more than 70% of the eukaryote proteome^9,10^. These identical or different domains are linked together by disordered segments characterized by variable lengths and sequences, also termed linkers. Due to their inherent variable flexibility, linkers can confer large conformational rearrangement to proteins to induce intra- or inter-domain interactions^11,12^. Therefore, MDPs possess the characteristics of both intrinsically disordered and well-folded proteins and their linkers can undergo post-translational modifications^13^ rendering their study more intricate^14^. A wide range of approaches has reshaped our understanding of multidomain proteins, including FRET^15^, SAXS^16^, SANS^17^ or NMR^18^, either as an individual technique or in combination^19^. From these methods, NMR spin relaxation measurements are probably the most important source of information when dealing with multidomain proteins as the collected data carry dynamical information for different spin sites measured at different magnetic fields at various experimental conditions^20,21^. Boosted by hardware and force field improvement, multidomain protein dynamics has also been studied by all-atoms molecular dynamics or metadynamics that allow the exploration of longer trajectories and wider conformational space^22–24^. Despite many studies in the field, some questions are still pending and remain unanswered, or at least need further inquiry. For instance, what is the function of the linker flexibility and dynamics with respect to molecular recognition processes like allostery, cooperativity or avidity? To shed light on this dangling question we have chosen to explore the interaction of various STAM2 UIM-SH3 constructs with different binding partners. Indeed, STAM2 is part of the ESCRT-0 complex, which is the most upstream component of the ESCRT machinery involved in lysosomal degradation^25^. To do so, the proteins that are directed for degradation are first tagged by Lys63-linked polyubiquitin chains^26^. Here, STAM2 harbors three ubiquitin-binding domains (UBDs), namely the VHS, UIM and SH3 domains that recognize and interact with ubiquitin (Ub) moieties. In a previous work, we have delineated how these three domains interact with Ub and Lys63-linked diubiquitin chains (Lys63-Ub_2_). While the VHS-UIM construct cooperatively engages Lys63-Ub_2_^27^, the UIM-SH3 has demonstrated an exquisite capability to bind Ub, Lys63-Ub_2_, UBPY and AMSH through the UIM and SH3 domains^28,29^. In the current article, we have selected and engineered five different constructs of the UIM-SH3 (further denoted US-WT) dual domain protein with different linker lengths or domain deletion. By means of SAXS, we show that the truncation of the linker results in a decrease in the US-WT flexibility and the conformational space sampled by each of the constructs. We have also investigated the dynamical properties at the ps-ns time scale through NMR spin relaxation measurements and we show that the correlation times related to the UIM and SH3 domains are significantly different. Moreover, the gap between the correlation times associated with each domain decreases along with the linker length. Finally, these observations were complemented by titration experiments where we have compared the interaction of Ub and Lys63-Ub_2_ with all the newly designed constructs. Our results indicate that the binding affinity between Ub and the various US constructs is rather similar while the affinity between Lys63-Ub_2_ and the various US constructs experiences a drastic change when the length of the linker is shortened and may have further implication in the binding and molecular recognition between multidomain proteins involved in lysosomal degradation.

## Structural Modifications Induced by UIM-SH3 Mutations

**Figure 1:**
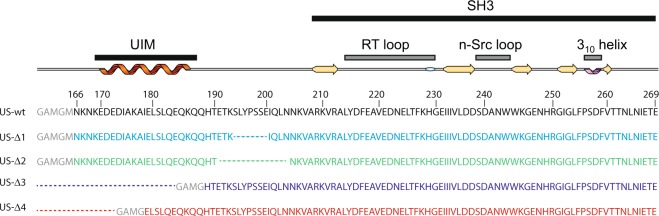
Name of the different constructs used in the present study along with their respective sequence and numbering. The schematic representation of UIM-SH3 secondary structure has been obtained by means of dssp^66,67^ embedded into cartoon representation program SSS-drawer (https://github.com/zharmad/SSS-Drawer).

To study the influence of the linker length on the flexibility and dynamical properties, we have selected and engineered US-WT derived variants where the tether that bridges UIM and SH3 has been modified in several ways. US-Δ1 and US-Δ2 have their linker shortened for 7 and 14 amino acids, respectively. While UIM was completely removed in US-Δ3 that leaves the SH3 domain only with the full-length linker, the C-terminal half of UIM remains in US-Δ4 (Fig. 1). As we have previously shown that the UIM and SH3 domains of the US-WT construct did not interact with each other, we have checked whether any mutation would have modified this structural hallmark. For each of the mutants, a ^1^H,^15^N-HSQC NMR spectrum was recorded and compared with the one obtained for US-WT. For US-Δ1, US-Δ2 and US-Δ3, the ^1^H,^15^N-HSQC spectra nicely overlap with the US-WT spectrum except for missing residues or residues located in the N-terminus of US-Δ3 (see Fig. S1). This result indicates that both UIM and SH3 in US-Δ1 and US-Δ2 keep a similar fold as UIM and SH3 in US-WT and that the linker shortening does not affect the UIM helix motif. Furthermore, the SH3 domain structure in US-Δ3 is not affected by the complete UIM deletion. Like US-Δ3, the US-Δ4 ^1^H,^15^N-HSQC spectrum corresponding to the SH3 domain nicely overlaps with the US-WT spectrum while some of the signals associated with the remaining UIM residues are shifted. This is likely due to a partial unwinding of the UIM helix after the cleavage of the US-WT N-terminus. Moreover, our circular dichroism results on US-Δ4 shows about the same helicity percentage than US-Δ3 while having a longer sequence (see Fig. S2 and Table ST1), which corresponds to a greater average number of helix residues (~5.7 for US-Δ4 over ~3.4 for US-Δ3). Furthermore, we can also exclude any transient interaction of the remaining UIM with SH3. To allow for a further analysis, backbone resonance assignment of US-Δ4 has been carried out by using a combination of the HNCO, HN(CA)CO, HNCACB and HN(CO)CACB experiments (see methods).

### Characterization of the flexibility of the UIM-SH3 constructs

**Figure 2:**
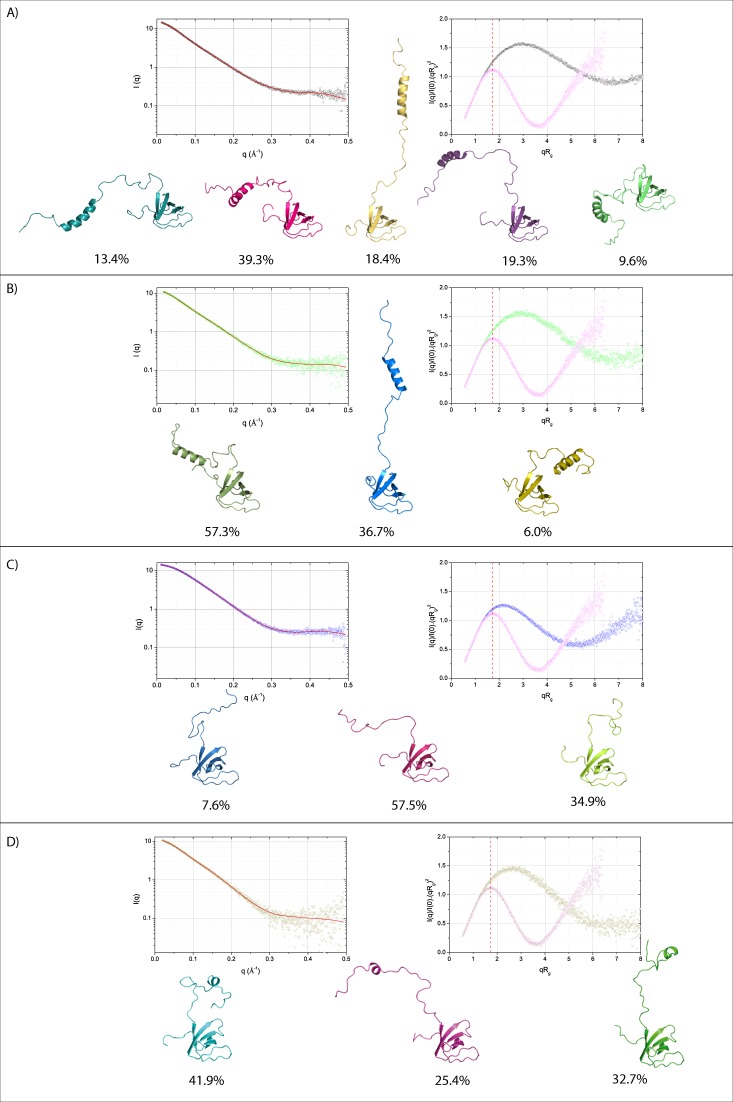
SAXS data (left) and dimensionless Kratky plots (right) for (**A**) US-WT, (**B**) US-Δ1, (**C**) US-Δ3 and (**D**) US-Δ4. A comparison with ubiquitin is given for each Kratky plot (pink). A dashed red line is plotted for a qR_g_ value of $\sqrt{3}$. It represents the typical peak maximum for globular proteins and is consistent with ubiquitin’s Kratky plot curve. The representation of the top-scoring N-ensemble of structure and their respective weight that best fit the SAXS data is presented at the bottom of each panel and are determined by means of the Multifoxs server^64^. The back-calculated SAXS data are represented by a continuous red line.

Thus, we were seeking to understand if any modification of the US-WT sequence would induce a drastic change of its flexibility and spatial organization. To this end, we have carried out SAXS experiments on the US-WT, Δ1, Δ3 and Δ4 constructs only. For all of them, data have been recorded at four different concentrations to ensure good quality data at both low and high angles (see methods). The SAXS data are presented in Fig. 2. Overall, the scattering profiles are smooth compared to a multidomain protein with fixed domain distances and reflect interdomain motion or a dynamic averaging^16^. Further Guinier analysis provides the radius of gyration (Rg) values and information on the average size of the different constructs in solution. On average, US-Δ3 displays the shortest R_g_ while other constructs have R_g_ close to each other (see Table ST2). To characterize protein flexibility, one frequently resorts to a so-called Kratky plot, which highlights the difference between a well folded, compact protein with a pronounced curve maximum and a random, unstructured chain that exhibits a plateau at the same qR_g_ value^30^. However, this kind of graphical representation is difficult to compare between objects of different size or different multidomain proteins. Therefore, we have represented dimensionless Kratky plots (Figs 2 and S3B) where I(q) is normalized to the forward scattering intensity I(0) and q is normalized to R_g_. Indeed, a typical fully folded globular protein (here exemplified by ubiquitin on Fig. 2) shows a curve maximum of ~1.104 at a qR_g_ value equals to $\sqrt{3}$ (~1.73) and a shape that rises and falls symmetrically^30,31^. Opposite to this situation, a random chain would exhibit a nearly hyperbolic curve followed by a further increase at higher q values. In the case of all the US variants, the dimensionless Kratky plots display a significant deviation from both the ‘ideal’ folded case and the classical disordered case with a non-symmetrical curve, a shift of the curve maximum and a slow decrease at higher q values (Fig. 2). This behavior confirms that each US construct contains at the same time well-folded and intrinsically-disordered region. Moreover, US-Δ3 shows a curve maximum for qR_g_ closer to 1.73 which reflects a lower composition of unstructured flexible regions compared to the other three constructs (Figs 2C and S3B). According to the dimensionless Kratky plot, it is also noticeable that the curve depicting US-WT falls with a less pronounced slope at higher q values. Thus, it is likely that the US-WT construct is the one affected by the highest flexibility among other variants and that the shortening of the linker has removed part of the contribution of the flexible disordered region. The P(r) functions present an asymmetric shape with a maximum around 17.5 Å (Fig. S3A). For the four constructs, this peak at low r value corresponds to intradomain distances that mostly arise from SH3, the biggest among the two domains. After reaching a maximum, the P(r) curves decrease in a bumpy manner with slightly pronounced humps. This behavior firstly corresponds to the presence of the second domain UIM, that is a small helix (for US-WT, US-Δ1 and US-Δ4) and secondly reflects the possibility for the different constructs to adopt various spatial conformations due to their flexible linker. US-WT, US-Δ1 and US-Δ4 have a similar pattern and differ only by the extent of their curve at high r values. US-Δ3 adopts a more symmetric curve that reflects a more compact structural organization in solution. Further analysis of the P(r) function allows the determination of D_max_ that corresponds to the maximum diameter of the protein. The US-WT possesses the highest D_max_ and samples the widest volume among all the different constructs (Table ST2). A shortening of the linker region by seven amino acids does not display a significant effect on D_max_ and R_g_. This result indicates that: (i) Despite the presence of seven more amino acids, the maximum diameter of US-WT is close to US-Δ1, therefore its linker does not behave as a fully elongated tether but rather shows turns and loops. (ii) Regardless of its shorter sequence length, US-Δ1 still occupies a significant volume and displays an average size comparable to US-WT. Further deletion of the US-WT N-terminus by 14 amino acids (US-Δ4) induces the partial deletion of UIM but only leads to a marginal change of R_g_ while D_max_ drops by 11 Å to adopt a shorter maximum diameter compared to US-WT. Conversely, one can notice a drastic change after the complete deletion of the UIM domain (US-Δ3) where both R_g_ and D_max_ show a significant decrease of 4.6 and 25 Å respectively. Therefore, these results support the fact that US-Δ3 has a more compact organization along with a reduction of the explored conformational space. These findings support the idea that the UIM domain acts as a spring that can maintain the SH3 domain at a given distance. To characterize the conformation adopted by the different constructs, we have used the ten modeled structures generated by Modeller (see methods) as starting structure for SAXS data fitting. SAXS data were back-calculated by means of the FoXS program^32,33^ and none of the initial models have succeeded to reproduce the SAXS curves and have given a systematic high χ score (see Table ST3). Thus, it is unlikely that the different US constructs exist in a single conformation. Therefore, we used MultiFoXS for computing N-state (N = 1 to 5) models of the US constructs and saving the 1000 top scoring after starting from 10000 initial conformations generated through the RRT algorithm (see methods). For the US-WT construct, the χ score significantly decreased with a one state model compared to the best model issued from Modeller (from 101.00 to 1.62). A further increase of the number of conformations to five states improves the χ score by around 50% and decreases the residuals (see Table ST3 and Fig. S4A). This result underlines the fact that different conformations co-exist in solution, from an elongated to a compact state along with different intermediate states. The number of states is also confirmed by the analysis of the R_g_ distribution computed for the 1000 best-scoring N-state models (Fig. S5). The R_g_ distribution in the initial pool of 10000 conformations is almost uniform and span a large amplitude from 16 to 35 Å. Whatever the model, a highly populated R_g_ is found around 20 Å while four other values of R_g_ appeared for two to five-state models. For the five-state model that shows a significant improvement of the χ score, three values of Rg are approximately homogeneously populated at 33.8, 26.8 and 24.8 Å while a lowly populated value of Rg appears at 15.8 Å. These results demonstrate that US-WT is sufficiently flexible and dynamic to explore a broad conformational space. For the US-Δ1 construct, the χ score significantly decreased with a one state model compared to the best model issued from Modeller (from 25.57 to 1.39). Moreover, the SAXS data are nicely reproduced with an optimum solution using a three-state models compared to the one-state model with an improvement of 28% (see Table ST3 and Fig. S4B). A four or five-state model did not improve the quality of the calculated SAXS data. As for US-WT, the one-, two- or three-state models share a common value of R_g_ that represents a more compact structure of US-Δ1 (R_g_ = 19.6 Å for the three-state model). All these models also show two other values of R_g_ and reinforce the choice of a three-state model for the US-Δ1 construct (R_g_ equals 28.6 and 30.6 Å for the three-state model). For the US-Δ3 and Δ4, the lowest χ scores obtained for the best models supplied by Modeller are significantly higher compared to the one-state model (55.60 compared to 1.29 and 4.17 compared to 1.73 respectively). Furthermore, we have retained an optimum three-state model that improves the χ score by 33 and 39% compared to the one-state model for US-Δ3 and Δ4 respectively (Table ST3). None of the four- or five-states model further improve the calculation of the SAXS curves (Fig. S4C,D). The R_g_ distribution related to the US-Δ4 is spread on a region comparable to the US-Δ1 construct and is consistent with the fact that the experimental R_g_ for US-Δ1 and US-Δ4 are quite similar despite a different sequence. For US-Δ4, the three-state model shows two highly populated R_g_ values at 15.7 and 20.7 Å that correspond to compact structures and two lowly populated R_g_ at 22.7 and 25.7 Å that correspond to a more extended conformation. As opposed to the US-Δ4, the R_g_ distribution associated with US-Δ3 spans a narrow region between 13 and 21 Å with three main population of R_g_ at 15.2, 18.2 and a lower populated R_g_ at 20.2 Å showing that its conformational arrangement stays rather compact. Indeed, this result is in good agreement with the R_g_ derived from a Guinier analysis. Overall, the N-state model analysis provides a more accurate and informative view of the different US construct compared to the common experimental R_g_ value that represents an average value over all possible conformations. It shows that the conformational space sampled by the different US construct could be described by a combination of different R_g_ along with their respective weight and delineates the dynamics of the current US mutants. While the comparison of the experimental R_g_ did not show any dramatic difference between US-WT, US-Δ1 and US-Δ4, the N-state model reveals a more contrasted depiction of the different conformations adopted by the various US constructs with maximum R_g_ values of 33.8, 30.6, 25.7 and 20.2 Å for US-WT, US-Δ1, US-Δ4 and US-Δ3 respectively.

### Dynamical properties in the ps-ns time scale of the UIM-SH3 variants

**Figure 3:**
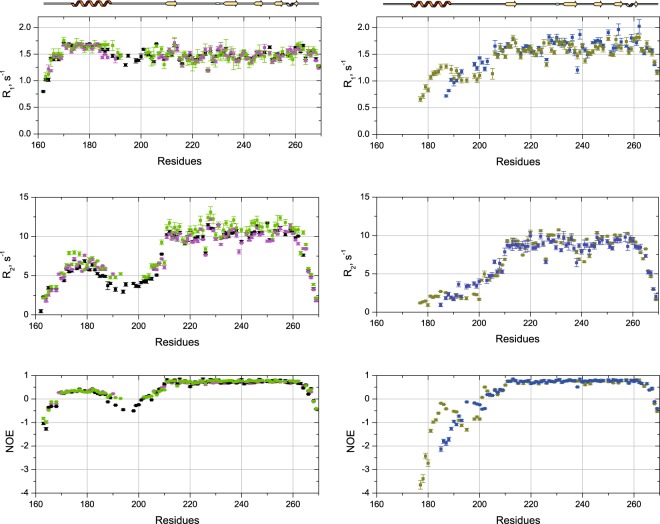
Relaxation parameters ^15^N R_1_, ^15^N R_2_ and ^15^N-^1^H heteronuclear NOE for the different US constructs according to the following color code: US-WT (black), US-Δ1 (green), US-Δ2 (magenta), US-Δ3 (blue) and US-Δ4 (dark yellow). The differences in the R_1_ and R_2_ levels for SH3 and UIM indicate that they tumble with different correlation times. Negative and close to zero heteronuclear NOE values also indicate a high degree of backbone flexibility in the intervening linker as well as in the N and C termini of the different constructs.

While the global flexibility of the different constructs has been investigated through the measurement of SAXS data, a more accurate description of the dynamics is necessary to characterize the different time scales (in the ps-ns range) that affect the various UIM-SH3 mutants. As we have noticed that a deletion of 7 amino acids in the linker region has a consequence on the flexibility of UIM-SH3, we have engineered a US-Δ2 construct where 14 amino acids have been deleted in the linker area (see Fig. 1). We have investigated the ps-ns time scale molecular motion of the different US constructs through the measurement of the three commonly used spin relaxation parameters ^15^N-R_1_, ^15^N-R_2_ and the ^15^N-^1^H cross-relaxation rates, via the steady-state ^15^N{^1^H}NOEs (see methods). At a first glance, several observations can be drawn for the constructs that contain both a UIM and a SH3 domain. First, the UIM and the SH3 domains exhibit significant differences of their average R_2_ and NOEs (see Fig. 3 and Table ST5) while the profile of R_1_ values is rather flat. Second, if one considers that no conformational exchange affects either the SH3 or the UIM domain, their average R_2_ levels deviate significantly from what would be expected when taken individually (see Fig. S6). In this representation, the molecular mass dependence of R_2_ was utilized as a “molecular ruler” (calibrated using R_2_ data for UIM, Ub and Lys63-Ub_2_). For instance, the UIM individual domain shows an average R_2_ of 5.1 ± 0.6 s^−1^ for an expected molecular mass of ~4.0 kDa^27^. Thus, our ^15^N relaxation data clearly support the fact that the UIM and SH3 domains involved in the different US mutants, tumble differently but not independently. Moreover, it is likely that UIM and SH3 do not share a common rotational diffusion tensor. The interdomain motions drastically affect the global shape of the protein and hence its rotational diffusion, anisotropy and global tumbling. Additionally, if one considers a 36% correlation between domains reported in a previous work of Bae et *al*.^34^, it corresponds approximately to the increase in R_2_ seen by the UIM and SH3 domain compared to the values expected for these domains taken individually. Therefore, we have chosen to derive the dynamics of the US mutants by considering a different rotational diffusion tensor for each of the domains. From the global tumbling analysis, it is noticeable that the differences between the correlation times that affect the UIM or the SH3 domains in US-WT, US-Δ1 and US-Δ2 are rather similar although US-Δ2 shows the shortest correlation time associated with the shortest linker. In the same order of idea, the gap between the average R_2_ for the UIM and SH3 domains significantly decreases when the length of the linker is shortened (see Table ST5) and clearly reflects the fact that the interdomain motion becomes less prominent when the linker is shortened. It is also noteworthy that the NOE values in the UIM C-terminus region for US-Δ1 and Δ2 exhibit higher values compared to the US-WT and support the conclusion that the linker shortening introduces a higher stiffness at the UIM C-terminus. This also lends credence to the fact that the domain coupling becomes less obvious when the linker length decreases below a given value. When the UIM is completely or partially deleted, the SH3 domain in US-Δ3 and Δ4 exhibits a significant decrease in its average R_2_ level with a concomitant increase in R_1_, consistent with a decrease in the apparent molecular mass. These observations are also supported by a drastic reduction of the correlation time of the SH3 domain (see Table ST5). For US-Δ3 and Δ4, the long flexible N-terminus displays much lower R_2_ and NOE values (up to −3.6 for NOE) compared to the same residue range in US-WT, Δ1 and Δ2. This observation clearly reflects much faster local motions and, therefore, a much higher flexibility of this part in US-Δ3 and Δ4. A more likely explanation can be given with the use of reduced spectral density mapping that makes no assumption about the nature of the rotational diffusion. R_1_, R_2_ and NOE can directly be expressed as a linear combination of spectral densities operating at three different frequencies J(0), J(ω_N_) and J(0.87ω_H_)^35^ (see supplementary materials). As previously discussed, these analytical expressions can also be used for multidomain proteins containing disordered segments^20^. The high frequency spectral density functions J(0.87ω_H_) are sensitive only to fast internal motions on a picosecond timescale while the zero frequency spectral density functions J(0) are sensitive to nanosecond internal motions that are faster than the global tumbling. As can be seen in Fig. S7, US-Δ3 and Δ4 are characterized by restricted motions in the SH3 core and exhibit large J(0) values while they clearly show high contribution of picosecond timescales (see range 180–200 on Fig. S7), and hence a much higher flexibility of their N-terminus part compared to US-WT, Δ1 and Δ2. As observed above, US-Δ1 and Δ2 display a reduction of the high frequency motion concomitant with an increase in the slow nanosecond motion of the UIM C-terminus region and hence, reflects a decrease in flexibility for the corresponding constructs.

### How molecular recognition is modified by the linker length and flexibility

As alluded to above, SAXS and NMR spin relaxation data have shown a difference in terms of rigidity and dynamics along with the length of the linker that bridges UIM and SH3. In a previous work, we have shown that US-WT has the capability to bind the deubiquitinating enzymes UBPY and AMSH^28^ as well as Ub and Lys63-Ub_2_^29^. Therefore, the next pending question could be “Do the length, rigidity and dynamics of the US-WT linker affect the interaction of US with its binding partners?”. To answer this question, we have monitored chemical shift perturbations (CSPs) of ^15^N-labeled US constructs upon addition of the corresponding unlabeled Ub and Lys63-Ub_2_ by means of ^1^H,^15^N HSQC experiments (Fig. S8). For US-WT, Δ1 and Δ2 that possess a complete UIM domain, several residues located either on the UIM and the SH3 domain experience significant CSPs or strong signal broadening. Since the UIM and SH3 domains do not interact with each other, any perturbation would result from a direct interaction of the US constructs with Ub. Overall, the CSPs patterns on the UIM domain for the different complexes are similar within each other while the number of CSPs above the threshold (fixed here at 0.4 ppm) for the SH3 domain slightly decreases, concomitant with a decrease in the US linker length (Fig. S8). On the SH3 domain, the affected residues cover a region mainly centered on the RT loop and the 3_10_ helix. On the other hand, residues K208, V207 and A206 located close to the RT loop exhibit CSPs increase that is likely due to the shortening of the linker. The quantitative analysis of the titration curves allows us to derive the corresponding dissociation constants. As seen in Table 1 and in Fig. S9, saturation of the UIM and SH3 domains in US-Δ1 and Δ2 occurs at a higher concentration of Ub and the dissociation constant slightly increases approximately by the same amount for UIM on the one hand and SH3 on the other hand when US-Δ1 and then US-Δ2 is considered (~1.5 and ~2.4 times respectively). These observations led us to conclude that the shortening of the linker has only a modest impact on US binding affinity when Ub is a binding partner. Additionally, half or entire deletion of the UIM domain completely abolished Ub binding on the UIM side while the SH3 is not affected and still shows a significant binding affinity (see Table 1) with a similar K_d_ compared to US-WT. This is consistent with a previous result that demonstrated how a US^I178E^ mutant completely abolished Ub binding on the UIM side^28^. The STAM2 protein, which plays an essential role in the endosomal sorting process, is known to have a preferential binding activity with Lys63-linked polyubiquin chains^26^. Thus, we were seeking to understand if any modification of the flexibility and dynamics of the US linker would perturb its binding affinity with a Lys63-Ub_2_ dual domain protein. We used a similar protocol as the one used for Ub binding and recorded CSPs along with the addition of Lys63-Ub_2_ (Fig. S10). Overall, the CSP pattern for the US-WT/Lys63-Ub_2_ complex is comparable to the one seen for the US-WT/Ub binding and does not present additional perturbed residues. Nevertheless, a closer look at CSP located on the UIM domain reveals an increase in the CSPs on the US-Δ2 N-terminus while residues Q185 and Q187 experience a drastic decrease in their respective CSPs compared to US-WT. The analysis of the titration curves reveals a shift of the saturation point for several residues, more pronounced for residues Glu218, Ala219 or Lys186 on both US-Δ1 and Δ2. While the dissociation constant seen on the UIM domain for the US-WT/Lys63-Ub_2_ complex is comparable to the one seen for the US-WT/Ub complex (Table 1), the affinity of the SH3 domain for Lys63-Ub_2_ strongly increases compared to Ub and suggests an avid interaction, as already underlined^29^. It is also noteworthy that the affinity of the SH3 domain decreases drastically by a factor of ~8 with the shortening of the linker (US-Δ2) while the dissociation constant associated with the binding of UIM with Lys63-Ub_2_ is not affected. Additionally, Lys63-Ub_2_ shows the tightest binding with SH3 when the whole UIM domain has been deleted (US-Δ3). These results clearly support the conclusion that the UIM domain in US-Δ2 has an inhibitory effect with respect to SH3. This effect may be due to a combination of a shorter linker, steric hindrance and an excluded volume constraint of UIM that reduces the conformational space available for the binding of SH3. Our results also demonstrate that multidomain proteins encourage avid binding with other multidomain proteins and that any significant modification in the linker region may affect the binding affinity of the complex.

**Table 1:** Summary of the different dissociation constants measured in the present study.

		K_d_ (µM)	K_d_ (µM)
		Mono Ub	Lys63-Ub_2_
US-WT	UIM	86 ± 31	78 ± 59
	SH3	267 ± 119	60 ± 28
US-Δ1	UIM	148 ± 60	137 ± 57
	SH3	394 ± 127	253 ± 163
US-Δ2	UIM	203 ± 91	99 ± 50
	SH3	669 ± 234	483 ± 196
US-Δ3		266 ± 96	39 ± 22
US-Δ4		158 ± 79	97 ± 49

In the case of interaction with Ub, dissociation constants were extracted using a 1:1 model for all constructs. In the case of interaction with Lys63-Ub_2_, dissociation constants were extracted for US-WT, US-Δ1 and US-Δ2 using a 2:1 model and for US-Δ3 and US-Δ4 using a 1:1 model (see methods section). Standard deviations are used as error estimates.

## Discussion

US-WT is a dual domain protein that contains two ubiquitin binding domains (UBDs) and is a component of the STAM2 protein. This part of STAM2 is fundamental in the sorting process of lysosomal degradation and recognizes various binding partners^28,29^. More specifically, STAM2 itself is a component of the ESCRT-0 complex that binds ubiquitin or polyubiquitin chains with a preference for Lys63- over Lys48-linked chains^26,36^. Furthermore, to maintain a constant pool of ubiquitin in the cell, polyubiquitin chains require cleavage by the specific enzymes UBPY or AMSH. The AMSH catalytic activity is enhanced when STAM is present and is due to a given structural organization that promotes the association of STAM, Lys63-linked polyubiquitin and AMSH^29,37,38^. As can be seen, STAM2 and more specifically the US domains should accommodate different multidomain proteins in a spatially and timely fashion. Thus, it is of prime importance to understand how these different processes relate to the inherent plasticity of UIM-SH3, the latter domains being connected by a disordered linker of 20 amino-acids. One can also wonder if the motional properties of the linker play a role with respect to molecular recognition with various binders. Our results provide a first glimpse on the ability of US-WT to accommodate different binding partners due to the structural and dynamical characteristics of its linker. Here we took a bold and direct approach of progressively reducing linker regions and beyond-starting from WT, partial linker deletion, partial UIM deletion and finally a complete UIM deletion. The SAXS analysis of the variants demonstrates an evolution of changes in R_g_ and D_max_, starting with subtle decrease and towards more drastic change when both domain and linkers were affected. Nevertheless, the sole consideration of the R_g_ derived from SAXS data is not sufficient to account for the inherent flexibility of the different US variants. Indeed, R_g_ represents an average over multi-state conformations and may hide a subtle structural dispersity of the different US variants. Therefore, we used a multi-state approach to account for the various possible conformations and their interconversion that describe the SAXS curves. As seen in the results section, the US-WT construct could be described by a five-state model that spans a wide range of Rg values from 15.8 to 33.8 Å. Conversely, the US-Δ1, Δ3 and Δ4 only need a three-state model to match the SAXS data, US-Δ3 being represented by the more compact conformations. To the light of our results, one can wonder why US-WT and US-Δ1 or US-Δ4 have close values of R_g_ and D_max_ even though US-Δ1 shows a linker region deleted by seven amino acids compared to US-WT. One possible explanation is the formation of loop region in US-WT that allows conformations as compact as for US-Δ1. The average Cα-Cα distance between residue Gln188 and Ala208 for the US-WT and US-Δ1 models, gives a value of 37.7 and 28.3 Å respectively, thus a difference of 9.4 Å when one expects a end-to-end distance of ~23.8 Å for an individual peptide of the missing sequence in US-Δ1^39^. This fact also lends credence to our hypothesis that the average R_g_ of US-WT is explained by the formation of loops present in different conformational states rather than several extended polymer chains. Additionally, the excluded volume due to the presence of the UIM and SH3 domains may also play a dominant role in the conformational space sampled by the different US constructs. Indeed, it is noteworthy that the excluded volume represented by the UIM or the SH3 domains prevents the flexible linker to adopt certain conformations (as illustrated by Fig. S11) in the case of US-WT, Δ1 or Δ4 but allowed in the case of US-Δ3^40^. It will also greatly decrease the number of conformations otherwise accessible to a chain and will increase its average dimension or the apparent stiffness of the linker as reported by recent Monte Carlo simulations^39^. This apparent stiffness could also induce loop formation at a time scale of ~12–20ns^41^ that is averaged on the SAXS time scale measurement.

A more specific quantitative analysis of the different US constructs motions reveals that the UIM and SH3 domains tumble with different correlation times. If the linker length is shortened from US-WT to US-Δ2, the correlation times associated with UIM and SH3 both decrease while we observe a more pronounced decrease for the UIM domain probably due to the difference of the domain sizes. Moreover, the gap between the R_2_ of UIM and SH3 tends to decrease as if UIM would be part of SH3. This is not unexpected as R_2_ mainly reflects the global tumbling of the US constructs. Thus, it is likely that the latter domains do not behave independently and that the linker acts like a spring. It restricts the global motion of both domains leading to an increase in their correlation time and a coupling of their tumbling. Other studies have focused on a significant increase in the linker length and have reported a decrease in the correlation between domains when the linker length increases^34^. A similar situation has been observed in the case of identical repeating domains where the correlation times are largely overestimated compared to a situation where each domain is considered in its individual state^42^.

Then, we have investigated the consequences of such flexibility and global tumbling on the interaction with Ub and Lys63-Ub_2_, two proteins that have been shown to interact with US-WT^28,29^. Modifications of the linker length slightly affect the binding of Ub with either UIM or SH3 by a factor of ~2.5. Half or complete deletion of the UIM domain do not affect the Ub binding capability of SH3 while half of UIM is not sufficient to bind Ub. The situation is more dramatic when Lys63-Ub_2_ is considered as a US binding partner. Indeed, the UIM domain does not suffer from a shortening of the linker and keeps a similar dissociation constant (see Table 1) while the SH3 domain exhibits a decrease in affinity by a factor of ~8 in striking contrast with its interaction with Ub. Overall, it appears that US-WT needs a fine-tuned linker length to enable a suitable avid binding with Lys63-Ub_2_ and to avoid steric clash that would reduce US-WT association or dissociation rate. Moreover, one can infer that the US-WT linker length, flexibility and dynamics have been designed to match with the end-to-end length of the Lys63-Ub_2_ and more generally with polyubiquitin-linked chains that are the preferred recognition signal for the lysosomal degradation. The linker is uniquely designed to provide a synergy between amino-acids composition, length, flexibility, dynamics and the environment conditions (pH, pressure, temperature for instance) associated with allosteric or cooperative effects. In the case of STAM2, one can hypothesize that the two different intervening linkers between VHS, UIM and SH3 have been adequately shaped to allow at the same time a fast switching between Lys63-polyubiquitin chains and AMSH that transiently interact^29^ for a rapid remodeling of the interaction map. This hypothesis is supported by our five-state model that shows a possible interconversion between a wide range of conformations for US-WT. Alteration of interactions with the polyubiquitin tag and deubiquitinating enzymes may cause a defect in the lysosomal degradation process and trigger further disease^43^. The question of the variability of the linker length with respect to molecular recognition is fundamental and previous studies on the Smurf2 systems have also demonstrated that a longer linker decreases the affinity^44^ or that the linker length of different GH5 cellulase variants affects their kinetic behavior^45^. Other examples can be found with the linker that joins the tandem UIM domains of human Rap80. The latter one acquires a helical structure when bound to Lys63-Ub_2_ and different lengths of the linker give rise to a decrease in affinity^46^. In this study, the tandem UIM can be regarded as a molecular ruler and highlights the importance of its length with respect to polyubiquitin-linked chains selectivity that can undergo various geometries and chain connections^47,48^.

Next, one can wonder if there is a relationship between flexibility, dynamics at the ps-ns time scale and binding affinity. Our results demonstrate that the length of the linker affects directly the global tumbling of both the UIM and SH3 domains and their binding capability with Lys63-Ub_2_. We can hypothesize that the outcome is a reorientation of the connected domains at a favorable rate to be consistent with the association/dissociation rate constant of any binding event. Furthermore, reduced spectral densities indicate a fast local motion located in the linker and its vicinity. This flexibility of the linker tends to reduce with its shortening, as evidenced by the higher values of R_2_ and the corresponding lower values of J(0.87ω_H_). The interdomain motion may involve large-scale rotations and translations as demonstrated by the different structures sampled to account for the SAXS curves and may involve high energetic barriers. The fast local motion added to the rotational diffusion may encode successive states that help reducing these barriers and expose pre-existing conformations to binding partners. Finally, it is clearly demonstrated that flexibility and dynamics of the linker region have a direct impact on molecular recognition of the STAM2 protein with respect to polyubiquitin chains. It has to be recalled also that the lysosomal degradation sorting process is carried out by a homodimer assembled with STAM and Hrs^49^. The latter one also possesses a VHS and a double-sided UIM domain that add another possibility of cooperative binding. Such a process involves the interaction of the VHS and UIM domains of STAM with Lys63-linked triubiquitin (Lys63-Ub_3_) while the SH3 domain of STAM would bind the SH3 binding domain of AMSH^37,38^. We can anticipate that a shortening of the different linkers of STAM would prevent the correct positioning of polyubiquitin chains and/or AMSH and thus precludes the right catalytic task. Indeed, preliminary results obtained on a VHS-UIM mutant where we have substituted 14 amino-acids of the linker by 14 prolines, show a complete deletion of cooperative effect between VHS-UIM and Lys63-Ub_2_. To conclude, we have shown that the length, flexibility and dynamics of the linker binding the UIM and SH3 domains of STAM2 are fundamental characteristics to accommodate different binding partners where STAM2 can be perceived as a multiple-ligand binding platform that acts at a given time and position to trigger the correct signal outcome. The unique plasticity of the linkers involved in STAM2 also contributes to the possibility of an active/inactive switch through ubiquitin intra-molecular binding^50^. From a more general point of view, the linkers included in multidomain proteins could also be the next generation of druggable target as their modification may reduce or completely abolish interactions.

## Methods

### Protein expression and purification

The human STAM2 constructs with truncation and mutation (Fig. 1) were designed in pETM60 plasmid with NusA and 6-His tag fused to the N-terminus under the regulation of a *lac* operon and have been purchased from Genecust. The plasmids were then transformed into *E*.*coli* BL21 GOLD (Milipore). Cells were grown in LB medium with 50 mg/l kanamycin or M9 medium supplemented with 1 mM MgSO_4_, 1 mM CaCl_2_, 6 mg/l Thiamine, 1% (v/v) trace element solution [5 g/l EDTA, 0.5 g/l FeCl_3_.6H_2_O, 5 mg/l ZnO, 1 mg/l CuCl_2_.2H_2_O, 1 mg/l Co(NO_3_)_2_.6H_2_O, and 1 mg/l (NH_4_)_6_Mo_7_O_24_.4H_2_O], 50 mg/ml Kanamycin and 1 g/l ^15^NH_4_Cl as sole nitrogen source for a uniform ^15^N labelling. For ^13^C-labelling, 2.5 g/l of ^13^C_6_-D-Glucose were used instead of ^12^C_6_-D-Glucose. The cells culture was grown at 37 °C to an A_600_ of 0.6–0.8 and the overexpression is induced by adding 1 mM IPTG. After 5 h of induction at 30 °C, cells were lysed in 50 mM Tris buffer, 250 mM NaCl, 10 mM Imidazole, 0.04%(v/v) β-mercapto-ethanol, 5%(v/v) Glycerol and 1 tablet of Complete^®^ protease inhibitors from ROCHE. The clarified cells lysate was loaded on a Ni-NTA Fast Flow column (GE Healthcare) equilibrated with 50 mM Tris-HCl (pH 7.8), 250 mM NaCl, 10 mM Imidazole, 1%(v/v) glycerol and 0.04%(v/v) β-mercaptoethanol. The bound protein was eluted with a 10–400 mM imidazole gradient. NusA and His_6_ tag were cleaved by TEV protease at 4 °C O/N and discarded by a second Ni-NTA column. Proteins were then purified by a Superdex 75 gel filtration column (GE Heathcare) equilibrated in 20 mM sodium phosphate buffer (pH 6.8) and 130 mM NaCl. The elution peak was desalted and concentrated in a Microcon concentrator tube with 3 kD cut-off. Ubiquitin and Lys63-Ub_2_ chains were prepared according to the previously published method^51^.

### Homology modeling of UIM-SH3 constructs

The 3D structure of the UIM part (if any) in the UIM-SH3 constructs was obtained by homology modeling following a methodology similar to the modeling of the VHS-UIM construct^27^. The amino acid sequences of STAM2-UIM and Vps27-UIM1 share 55% identity and 70% similarity. We used the UIM1 domain^52^ of Vps27 (PDB code 1Q0V) to model the structure of the UIM part of the UIM-SH3 construct, while the STAM2 SH3 domain (PDB code 1X2Q ) was used to model the SH3 part of the UIM-SH3 construct. Models were generated by using the Modeller program^53^ and by considering the linker region as flexible. After an alignment of the query and template sequences with Align2D, the UIM1 domain of Vps27 and the SH3 domain of STAM2 were used as input in Modeller. A total of ten structures were generated for the different UIM-SH3 constructs.

### NMR experiments

NMR experiments were performed on a Bruker Avance III operating at a ^1^H resonance frequency of 600 MHz (14.1 Teslas) equipped with a triple TCI cryoprobe. Sample temperature was set to 15 °C to be consistent with our previous studies on this system. The NMR samples have been exchanged into a buffer containing 20 mM sodium phosphate (pH 6.8), 10% D_2_O and 0.02% (w/v) NaN_3_.

For US-Δ4 construct, the backbone resonance assignment was carried out by using a combination of the following experiments: HNCO, HN(CA)CO, HNCACB and HN(CO)CACB.

Relaxation measurements including ^15^N longitudinal (R_1_), transverse (R_2_) relaxation as well as the ^15^N-^1^H heteronuclear cross-relaxation rates were performed using the previous published method^54^. NMR spectra were recorded with spectral widths of 2069 Hz in the ^15^N dimension and 9615 Hz in the ^1^H dimension. For the R_1_ experiments, we used relaxation delays ranging from 40 to 2400 ms with a recycling delay of 2 s. In the case of R_2_ experiments, we used relaxation delays from 8 to 224 ms with a recycling delay of 3 s. For heteronuclear NOE experiments, 2D spectra were recorded with and without presaturation of amide protons. The relaxation delay was set to 4.5 s in order to allow the bulk water magnetization to return as close as possible to the equilibrium state.

### NMR titration studies

The affinities of the different constructs interacting with Lys63-Ub_2_ or Ub were characterized using chemical shift perturbation (CSP). A series of ^1^H-^15^N HSQC were recorded for ^15^N-labeled UIM-SH3 constructs upon addition of non-labeled Lys63-Ub_2_ or Ub until saturation. To derive the corresponding dissociation constant, we analyzed CSPs by calculating the combined amide CSP as $\Delta \delta ={[({(\Delta {\delta }_{H})}^{2}+{(\Delta {\delta }_{N}/5)}^{2})/2]}^{1/2}$ where δ_H_ and δ_N_ are the chemical change in ^1^H and ^15^N dimension upon Lys63-Ub_2_ or Ub addition. Two models described by Varadan *et al*. have been used to extract dissociation constant^55^. In the case of US-Δ3 and US-Δ4, it was not possible to discriminate perturbations from either the distal or the proximal domain of Lys63-Ub_2_ on the SH3 domain. Therefore, we used a 1:1 model for US-Δ3 and US-Δ4 upon Lys63-Ub_2_ addition and for all US constructs upon Ub addition. The observed CSPs is a weighted average between the two extreme values corresponding to the free (Δδ = 0) and the bound state (Δδ = Δδ_bound_):

1 $$\Delta \delta =\Delta {\delta }_{bound}([{L}_{0}]+[{P}_{0}]+{K}_{D}-\sqrt{{([{L}_{0}]+[{P}_{0}]+{K}_{D})}^{2}-4[{L}_{0}][{P}_{0}]})/2[{P}_{0}]$$

In the case of US-WT, US-Δ1 and US-Δ2, we used a 2:1 model for which each domain is interacting with one unit of Lys63-Ub_2_ and the corresponding equation is:

2 $$\Delta \delta =\Delta {\delta }_{bound}(2[{L}_{0}]+[{P}_{0}]+{K}_{D}-\sqrt{{(2[{L}_{0}]+[{P}_{0}]+{K}_{D})}^{2}-8[{L}_{0}][{P}_{0}]})/2[{P}_{0}]$$

All NMR data were processed with NMRPipe^56^, analyzed with Sparky^57^ or CcpNmr^58^ and relaxation rates were extracted using Relaxfit^54^. The different correlation times were derived by means of the Rotdif software^59,60^.

### SAXS experiments

The measurements were performed at the ESRF BioSAXS beamline BM29 (Grenoble, France), using a 2D Pilatus detector at an X-ray wavelength *λ* = 1.008 Å with a standard single instrumental configuration (samples being automatically mounted to a capillary and 10 frames with 1 s exposure using the flow-through mode) at 20 °C. Data processing and reduction were performed using an automated standard ESRF beamline software (BSxCuBE)^61^ and PRIMUS^62^ while the overall parameters derived from SAXS data were processed with SCÅTTER^63^. Wild type UIM-SH3 and the truncated forms (US-Δ1, US- Δ3 and US- Δ4) were exchanged into a 20 mM Tris-HCl Buffer (pH 8.0) with 150 mM NaCl and 1 mM DTT. To eliminate any inter-particle effects, data were measured at 3–5 different concentrations (1, 2, 4, 8, and 10 mg/ml) and merged where needed. The final proteins were prepared at different concentrations in the range between ~0.5 and 10 mg/ml to obtain high quality data from both the low angle range (low protein concentration to accurately extract the radii of gyration) and from the high angle range (high protein concentration for an accurate solvent subtraction).

### MultiFoXS N-state modeling

In an effort to characterize the range of conformations consistent with the SAXS data for US-WT, US-Δ1, US-Δ3 and US-Δ4, we analyzed the distribution of R_g_ through MultiFoXS N-state modeling^64^. As an input, we have provided the pdb structure calculated by Modeller (see paragraph above) that gave the best χ score from FoXs modelling^32^. Flexible residues have been defined according to Table ST4 and 10000 conformers have been sampled. In the first step, MultiFoXS samples the input structures with a RRT algorithm^64^ that significantly improves the sampling efficiency compared to random sampling. As a second step, a SAXS profile is calculated for each sampled conformation. Finally, the 1000 top N-states models are sorted according to their χ values.

### Circular dichroism

The far-UV CD experiments were performed on a Chirascan circular dichroism (CD) spectrometer (Applied Photophysics, Ltd) using a cuvette with a path length of 1 mm at room temperature. The instrument parameters were set to a step size of 0.2 nm, a spectral bandwidth of 0.5 nm, a time-per-point of 1 s. The different samples were in 20 mM sodium phosphate buffer, pH 6.8 and were measured at a concentration of 5–10 µM. The CD spectrum of each sample was then subtracted to the buffer, normalized to their respective concentration and then converted to the mean residue molar ellipticity unit. The final spectra were deconvoluted using the CDSSTR algorithm^65^ with the reference set 7 available at DICHROWEB (http://dichroweb.cryst.bbk.ac.uk/html/home.shtml).

## Supplementary information

Supplementary information
