# Supplementary material for: Molecular recognition of ubiquitin and Lys63-linked diubiquitin by STAM2 UIM-SH3 dual domain: the effect of its linker length and flexibility
Source: Sci Rep. 2019 Oct 10;9:14645. doi: 10.1038/s41598-019-51182-0 (PMC6787221; doi:10.1038/s41598-019-51182-0)
Supplement: Supplementary file 1 — Supplementary information [file 41598_2019_51182_MOESM1_ESM.docx]

**Molecular recognition of ubiquitin and Lys63-linked diubiquitin by STAM2 UIM-SH3 dual domain: the effect of its linker length and flexibility**

Minh-Ha Nguyen, Marie Martin, Henry Kim, Frank Gabel, Olivier Walker, Maggy Hologne

**Supplementary Information**

**Supplementary Figure S1**: Overlay of ^1^H-^15^N-HSQC spectra of US-WT and US-Δ1 (A), US-Δ3 (B), US-Δ4 (C) and US-Δ2 (D). For residues present in both US-WT and US-Δ1 or US-Δ2 or US-Δ3 or US-Δ4, correlation peaks nicely overlap and support the fact that no structural changes and no transient interaction between domains occur between US-WT and the different mutants.

**
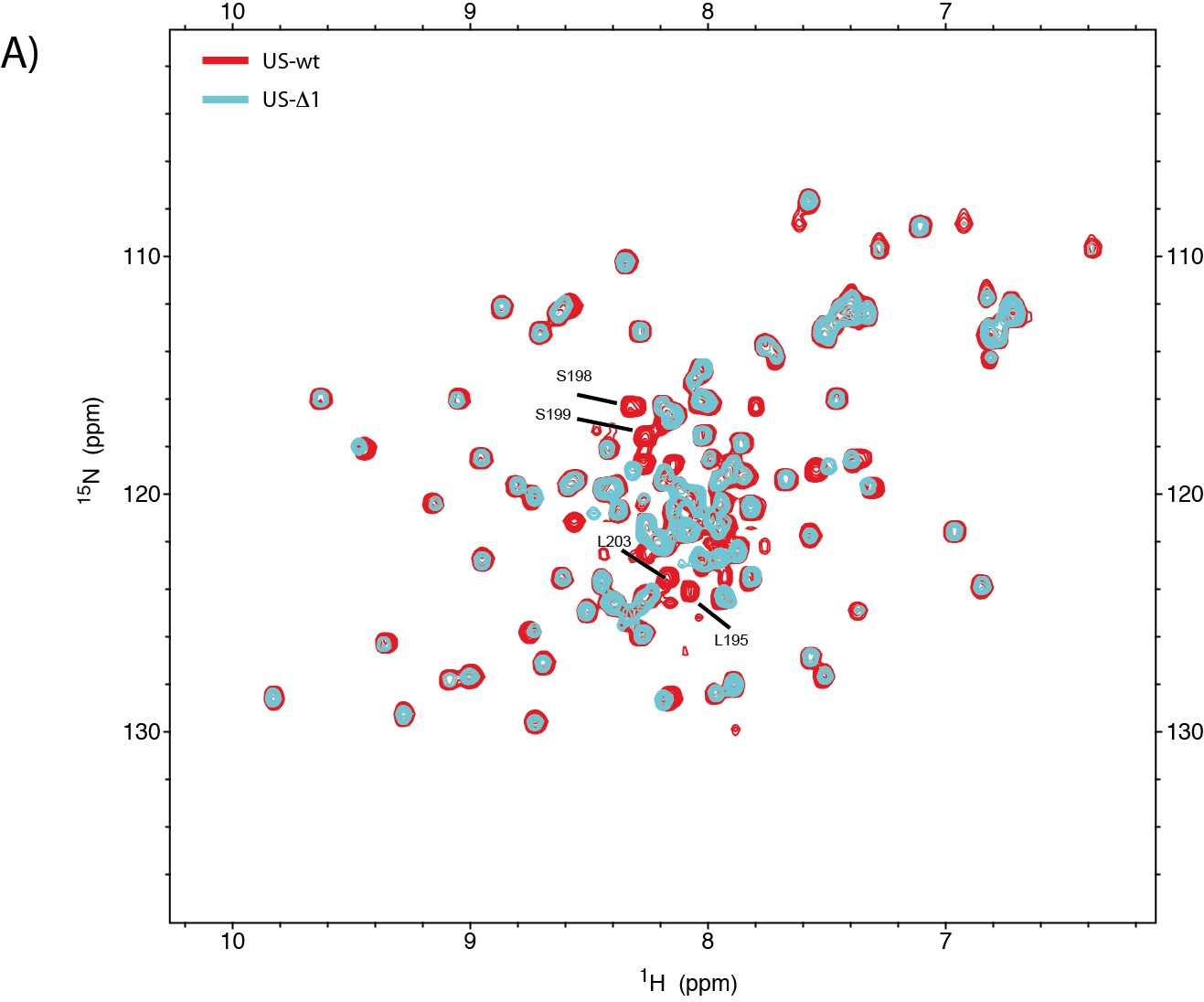
**

**
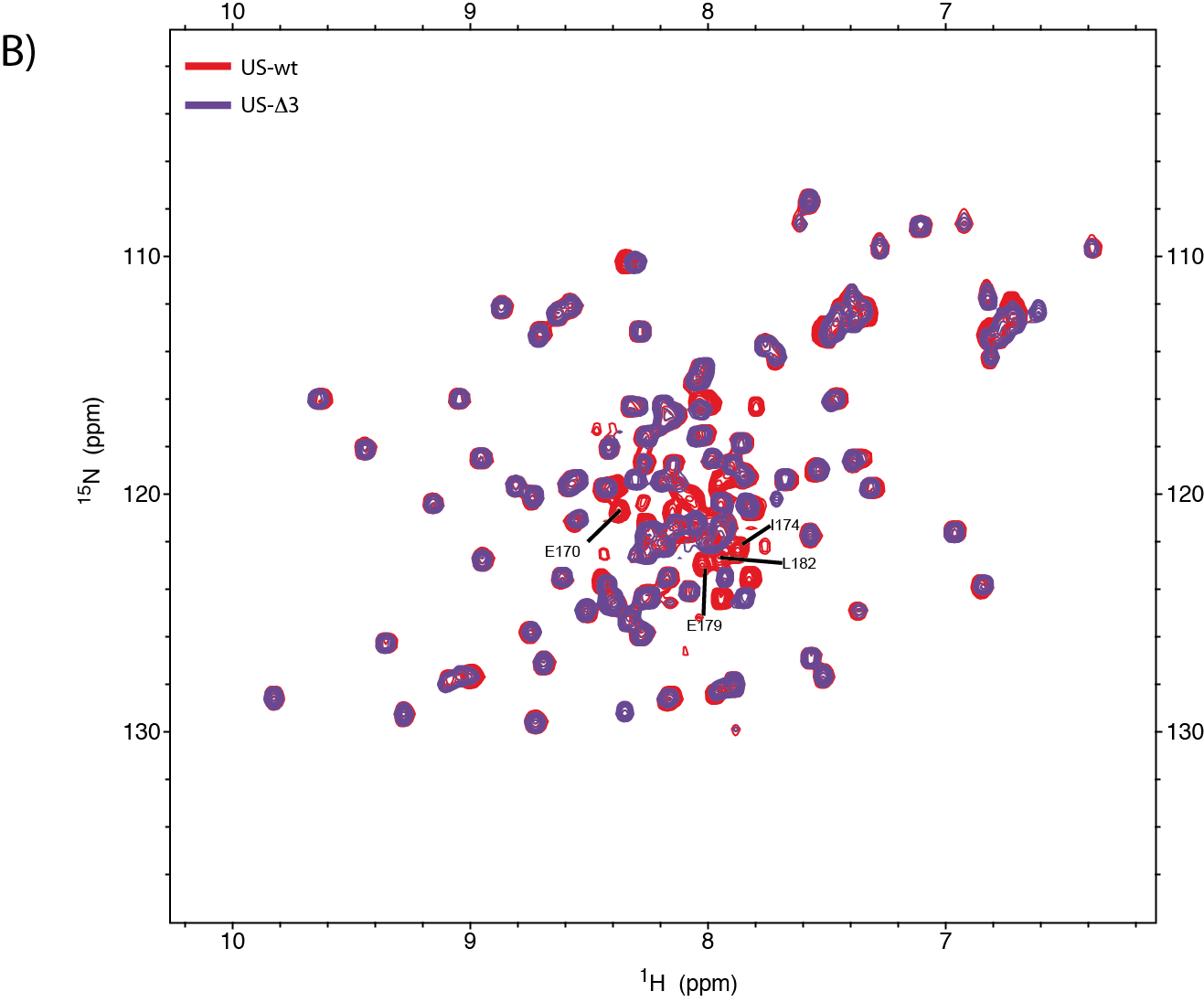
**

**
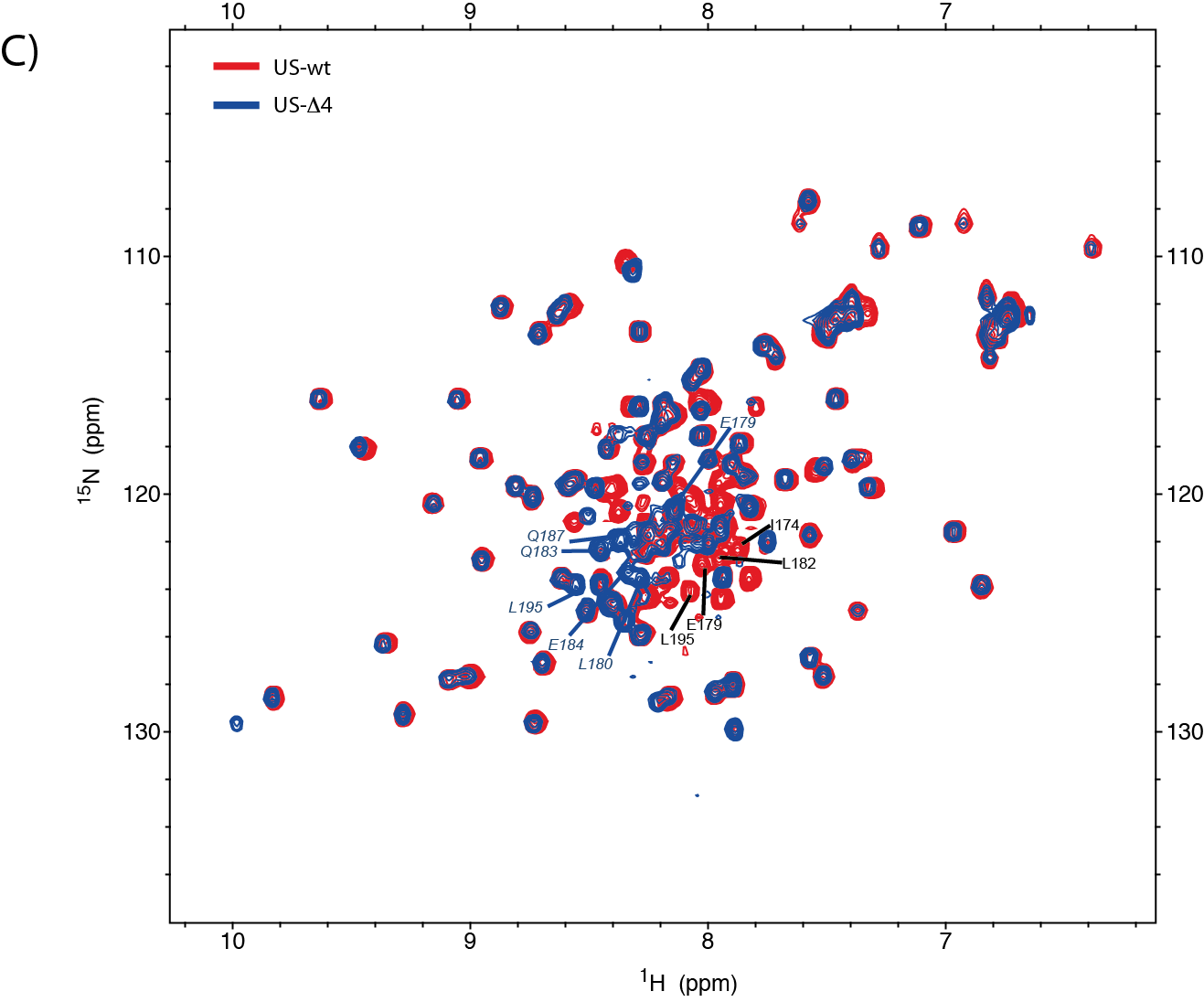
**

**
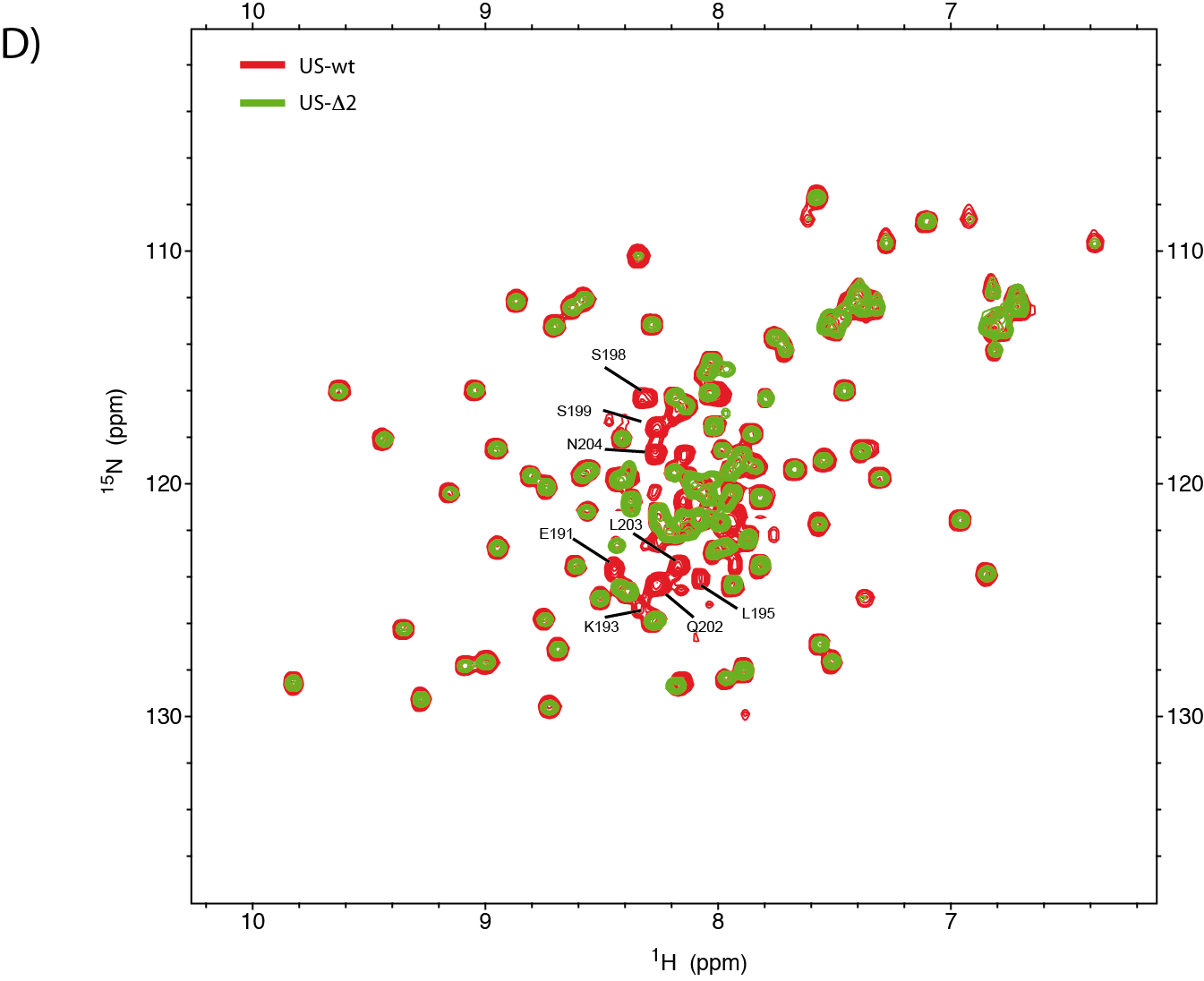
**

**Supplementary Figure S2**: Circular dichroism spectra obtained for US-WT (black), US-Δ1 (green), US-Δ3 (dark yellow) and US-Δ4 (blue). Experimental points are represented by open symbols while the fitted points are represented by red lines. The percentage of helicity (Table ST1) has been obtained by means of the Dichroweb server (http://dichroweb.cryst.bbk.ac.uk/html/home.shtml) and the CDsstr method^1^.


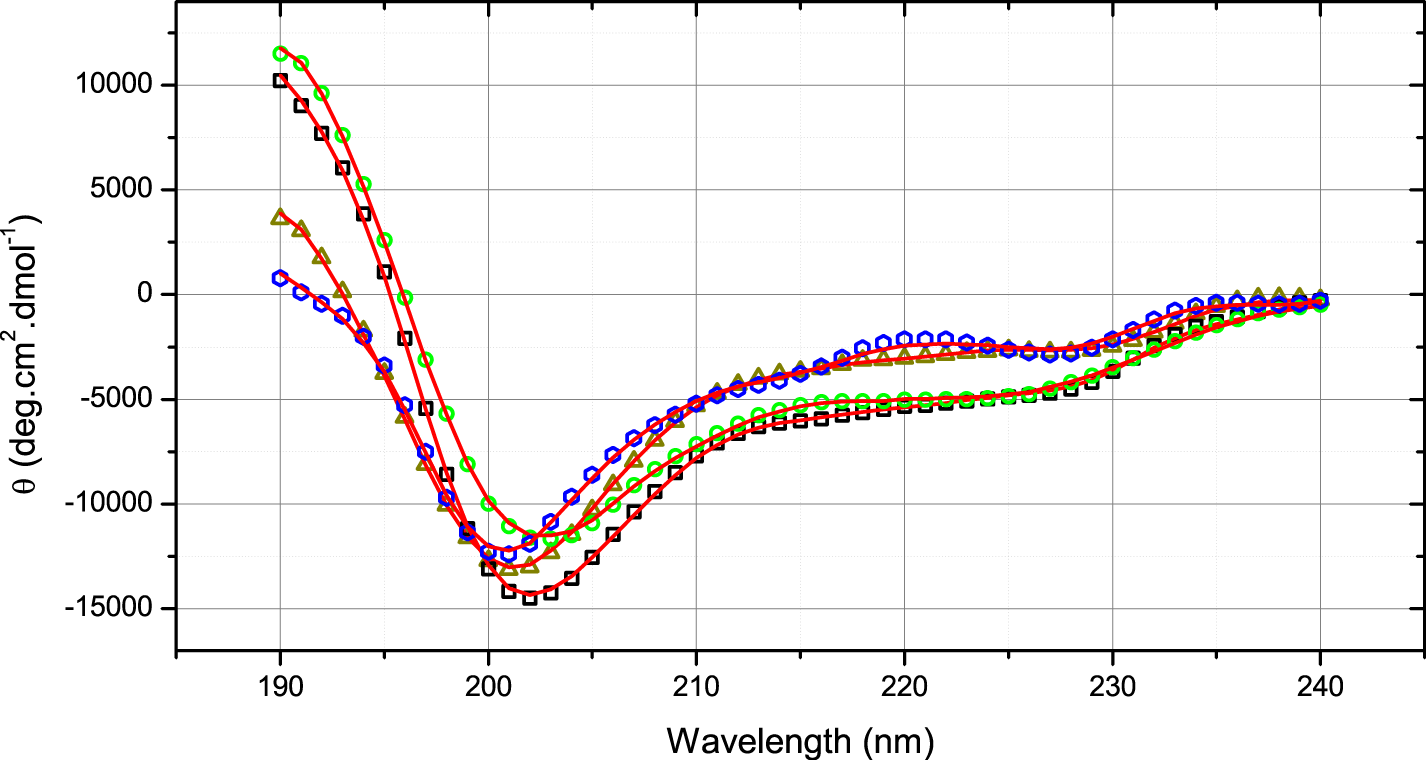


**Supplementary Table ST1:** Helicity percentage obtained with the data of Figure S2

| sample | helicity | Amino acids number |
| --- | --- | --- |
| US-WT | 16% | 109 |
| US-Δ1 | 16% | 102 |
| US-Δ3 | 4% | 85 |
| US-Δ4 | 6% | 95 |

**Supplementary Table ST2**: Overall parameters derived from SAXS data analysis. The radius of gyration (R_g_) was derived from a Guinier analysis while D_max_ was extracted from the distance distribution function P(r). The average volume of the different samples was derived with respect to the analysis of the Porod-Debye plateau. Analysis was conducted by means of the “SCÅTTER 3.1r” software^2^.

| sample | R_g_(Å) | D_max_(Å) | Volume(Å^3^) |
| --- | --- | --- | --- |
| US-WT | 22.13±0.26 | 95 | 21491 |
| US-Δ1 | 21.66±0.90 | 93 | 20919 |
| US-Δ3 | 17.48±0.09 | 70 | 17506 |
| US-Δ4 | 21.04±0.23 | 84 | 21304 |

**Supplementary Figure S3:** (A) Normalized distance distributions P(r) for US-WT (black), US-Δ1 (green), US-Δ3 (blue) and US-Δ4 (dark yellow). (B) Overlay of the normalized Kratky plots for each of the US mutants and Ub. Colors are similar as for panel A and Ub is colored pink. As a sake of comparison, the normalized Kratky plot of Ub has been added with the dotted line that represent a value of qR_g_ of $\sqrt{3}$, consistent with the typical peak maximum obtained for globular proteins.

| A) | B) |
| --- | --- |
|  | 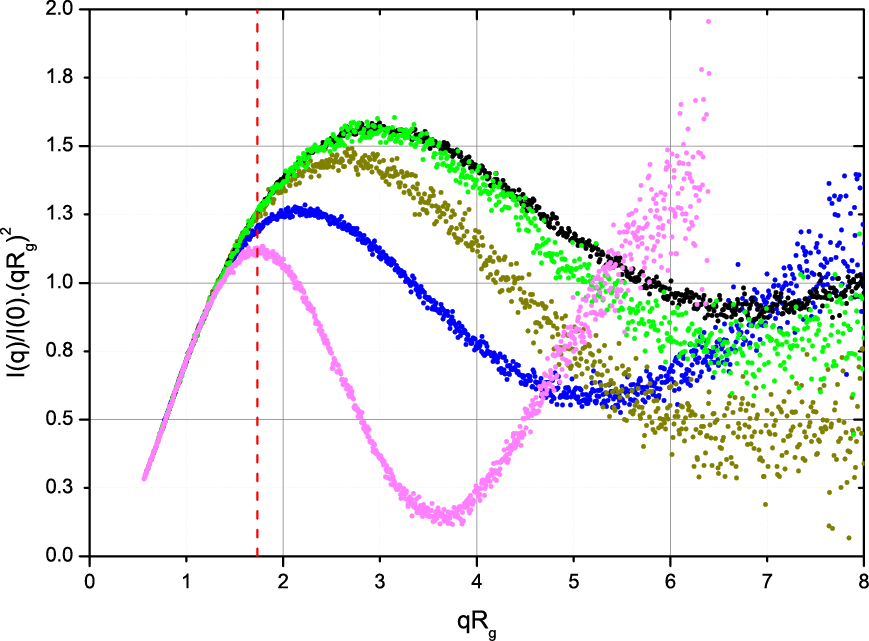 |

**Supplementary Table ST3:** Summary of the lowest χ scores for each of the different constructs and for the different N-state models used in MultiFoXS after the computation of 1000 conformations and starting from 10000 initial conformations. The highest χ scores for the top 1000 models is given into parentheses. As a sake of comparison, the lowest χ score calculated by FoXS for the 10 models generated by Modeller is also given. Note that the five-states model has not been computed for US-Δ4.

|  | US-WT | US-Δ1 | US-Δ3 | US-Δ4 |
| --- | --- | --- | --- | --- |
| 1 state | 1.62 (35.03) | 1.39 (37.43) | 1.29 (33.43) | 1.73 (53.44) |
| 2 states | 0.94 (1.46) | 1.05 (1.28) | 0.91 (1.44) | 1.25 (1.76) |
| 3 states | 0.90 (1.01) | 1.00 (1.04) | 0.86 (0.98) | 1.06 (1.29) |
| 4 states | 0.84 (0.90) | 0.98 (1.01) | 0.85 (0.92) | 1.08 (1.11) |
| 5 states | 0.79 (0.85) | 0.97 (0.98) | 0.85 (0.87) | N.A |
| Modeller | 101.00 | 25.57 | 55.60 | 4.17 |

**Supplementary Table ST4**: Range of the flexible residues used for the computation of the N-state model ensemble in MultiFoXS. The corresponding regions have been colored in red for the different structures.

| Construct | Flexible residues | structure |
| --- | --- | --- |
| US-WT | 161-170  189-207  262-269 | 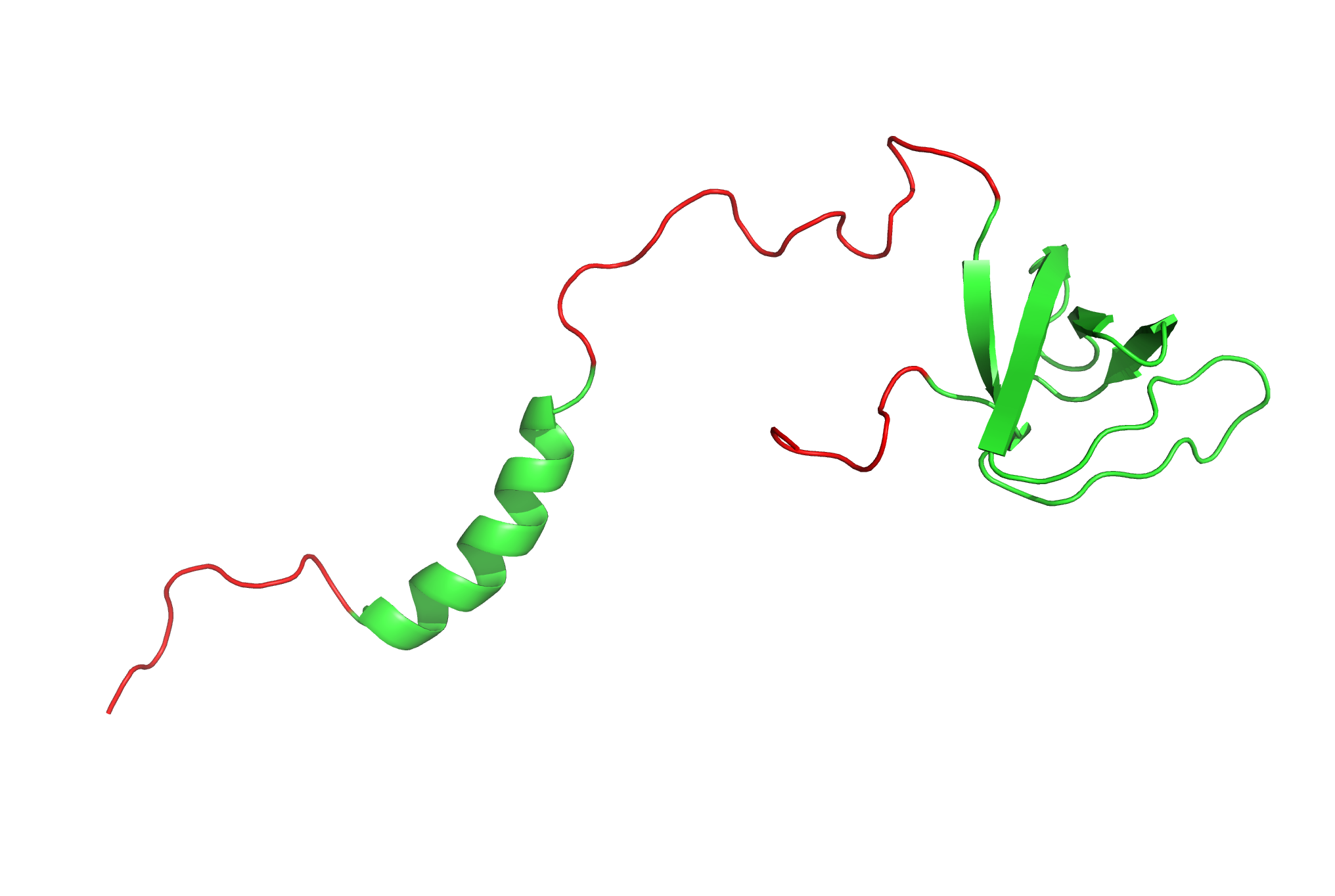 |
| US-Δ1 | 161-170  189-193  201-207  262-269 | 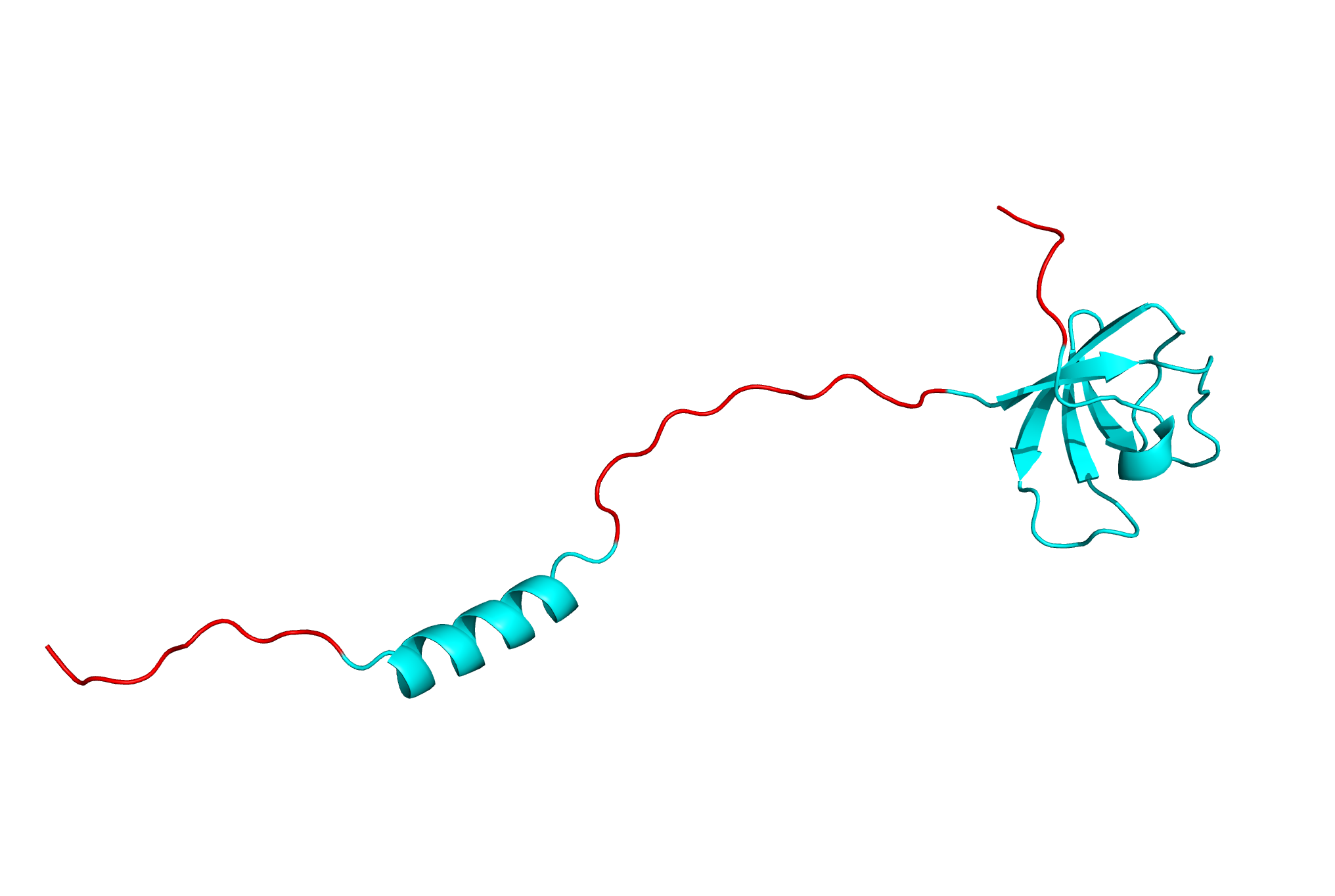 |
| US-Δ3 | 185-207  262-269 | 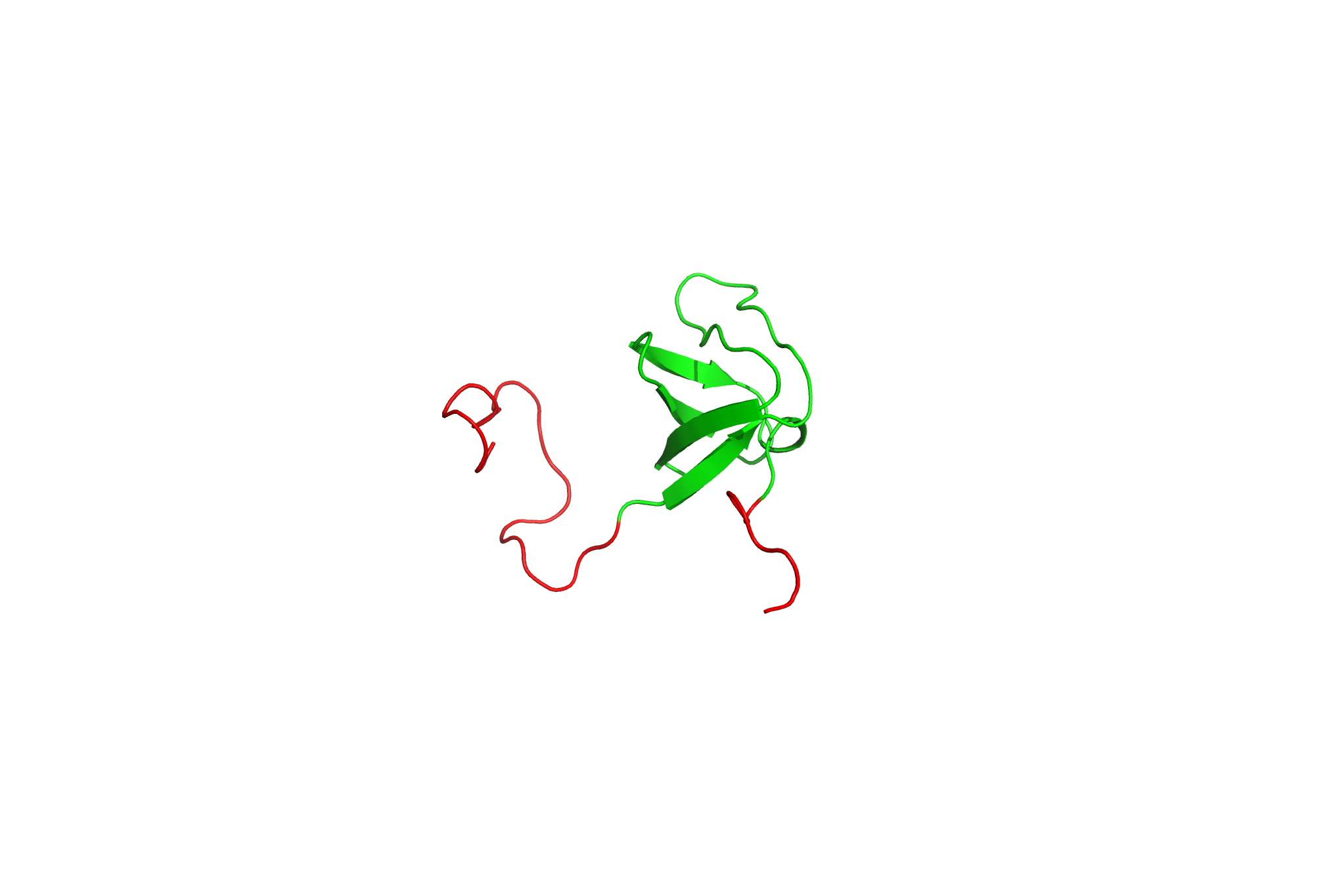 |
| US-Δ4 | 175-184  190-207  262-269 | 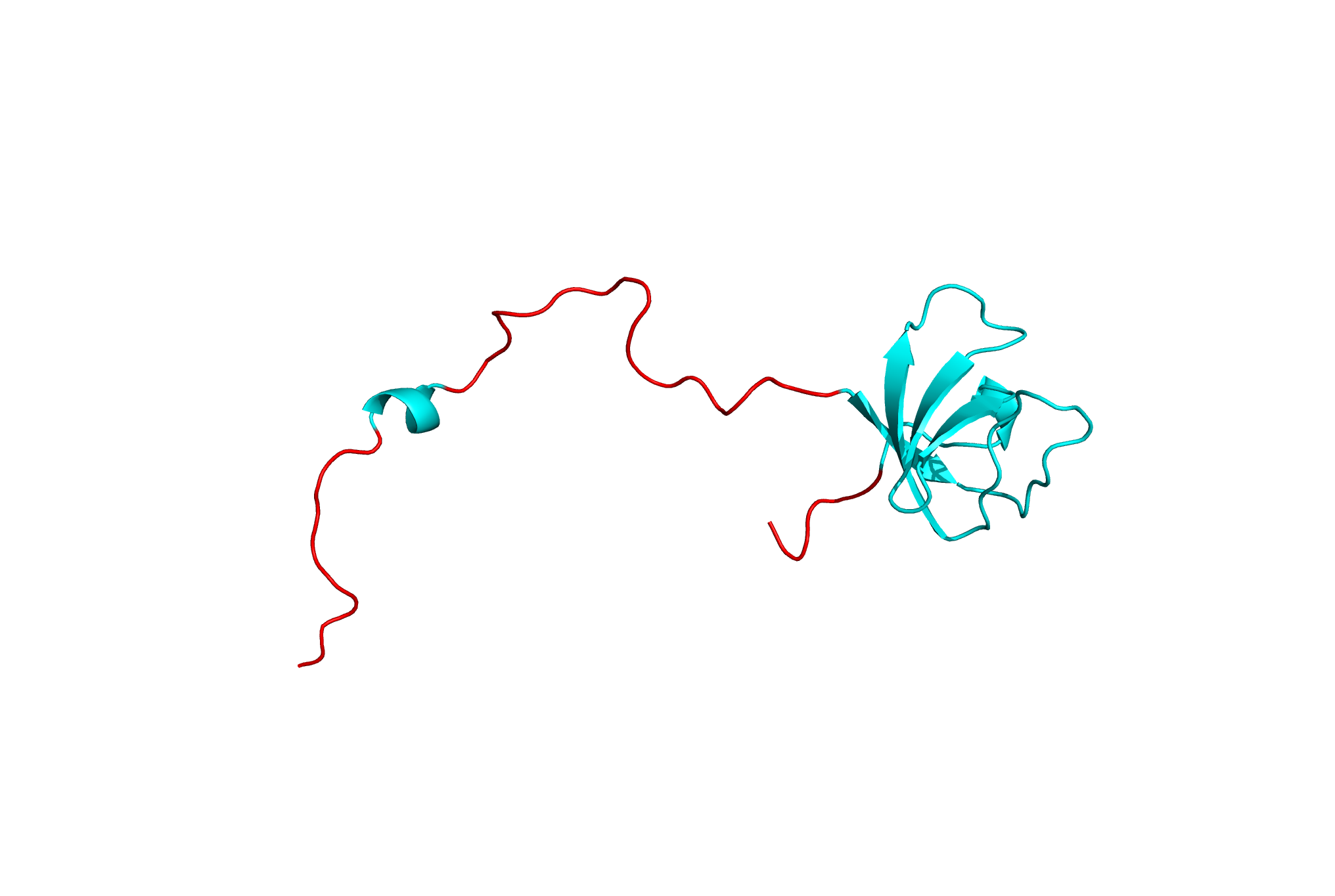 |

**Supplementary Figure S4:** Comparison of the experimental SAXS profiles (open circles) with computed SAXS profiles for the top-scoring single-state, the 3 or 5-state model and the top-scoring Modeller model calculated with FoXS^3^. The lower plots show the residuals defined as (I_exp_(q)-I_calc_(q))/σ_exp_(q), corresponding to the difference between the experimental and the computed intensities weighted by the experimental uncertainty. These plots are given for US-WT (A), US-Δ1 (B), US-Δ3 (C) and US-Δ4 (D).

**
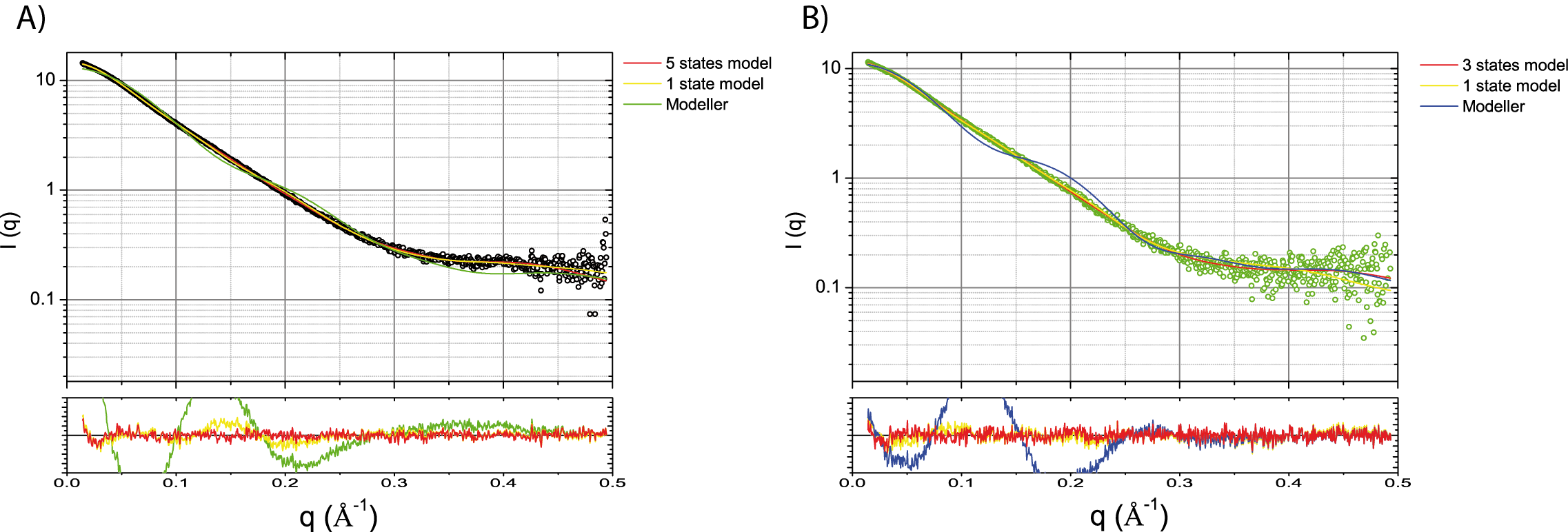
**

**
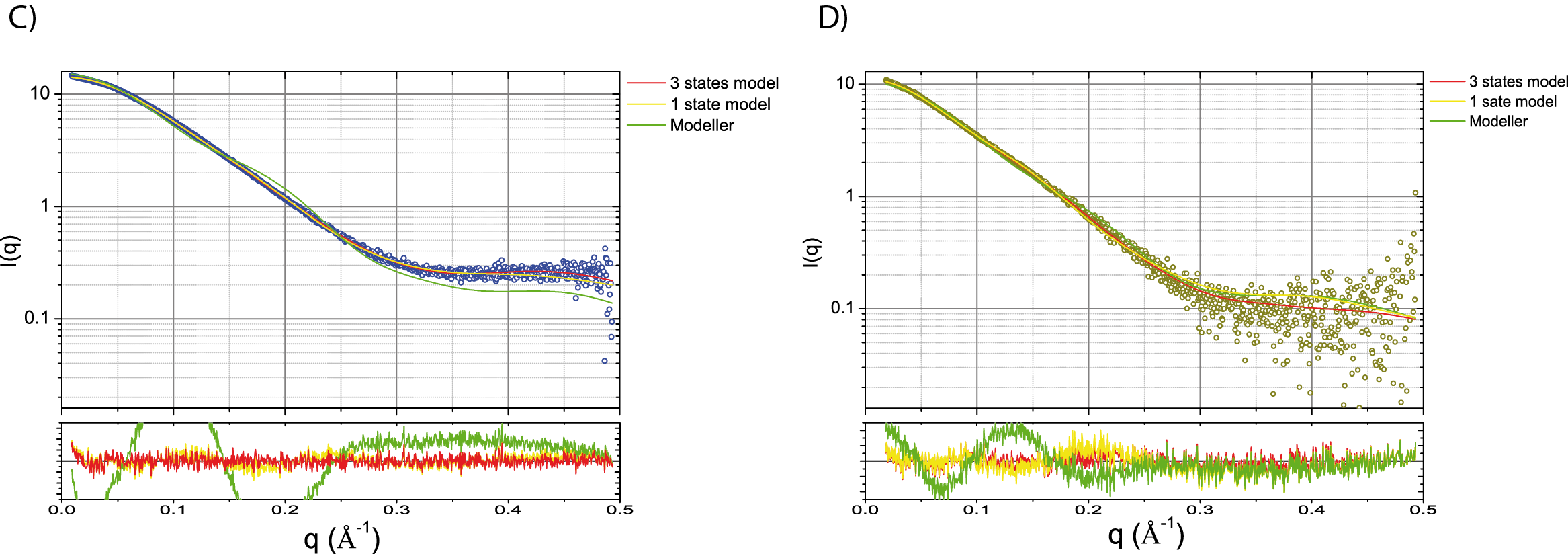
**

**Supplementary Figure S5:** R_g_ distribution for the entire ensemble of 10000 conformations (black lines) and the 1000 best-scoring N-state models (N=1 … 5) calculated by MultiFoXS (colored lines). Only the three-state models are shown for US-Δ1, US-Δ3 and US-Δ4 since increasing the number of states did not improve the χ score reported by MultiFoXS (see Table ST3).

| US-WT | US-Δ1 |
| --- | --- |
| 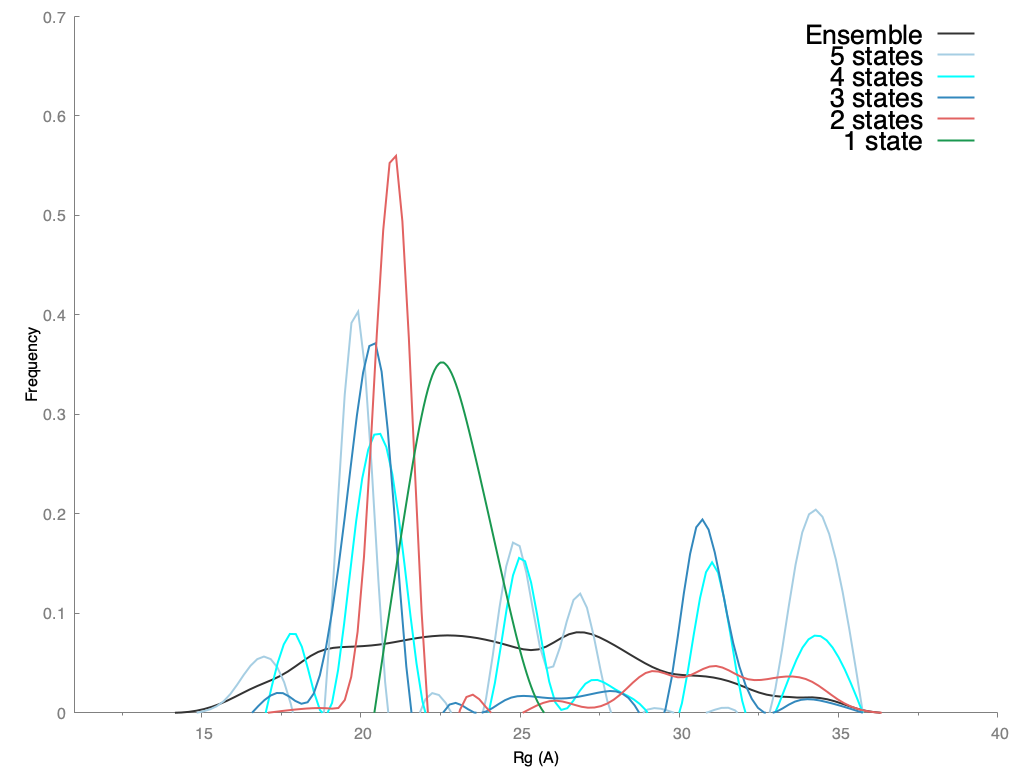 | 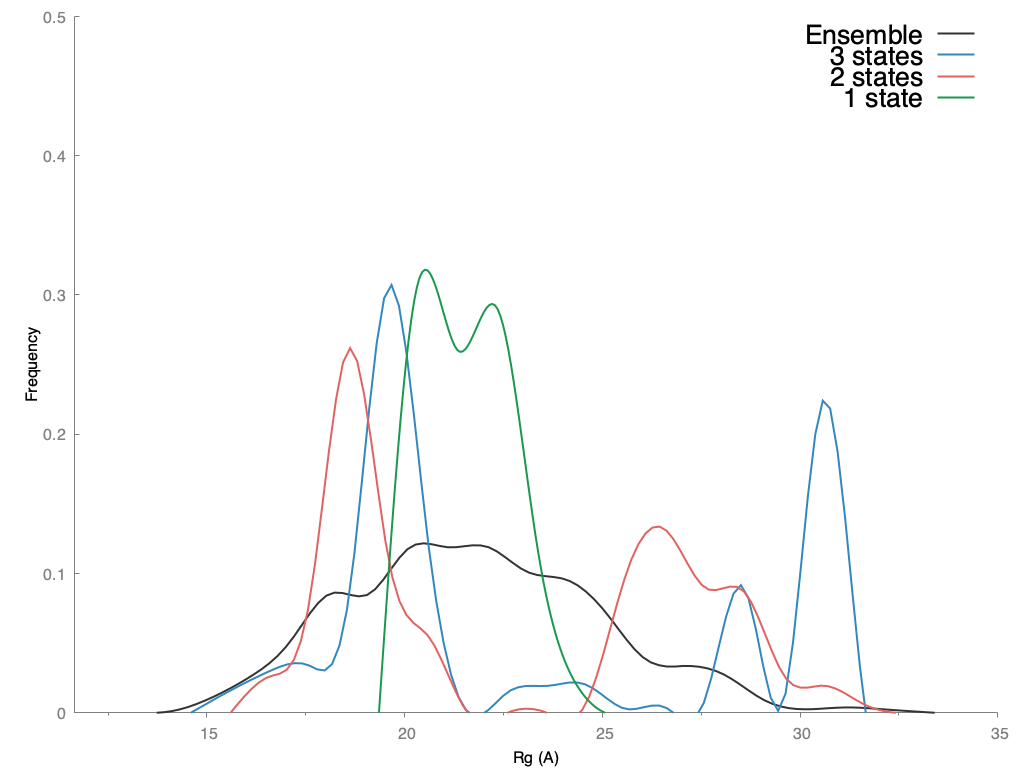 |
| US-Δ3 | US-Δ4 |
| 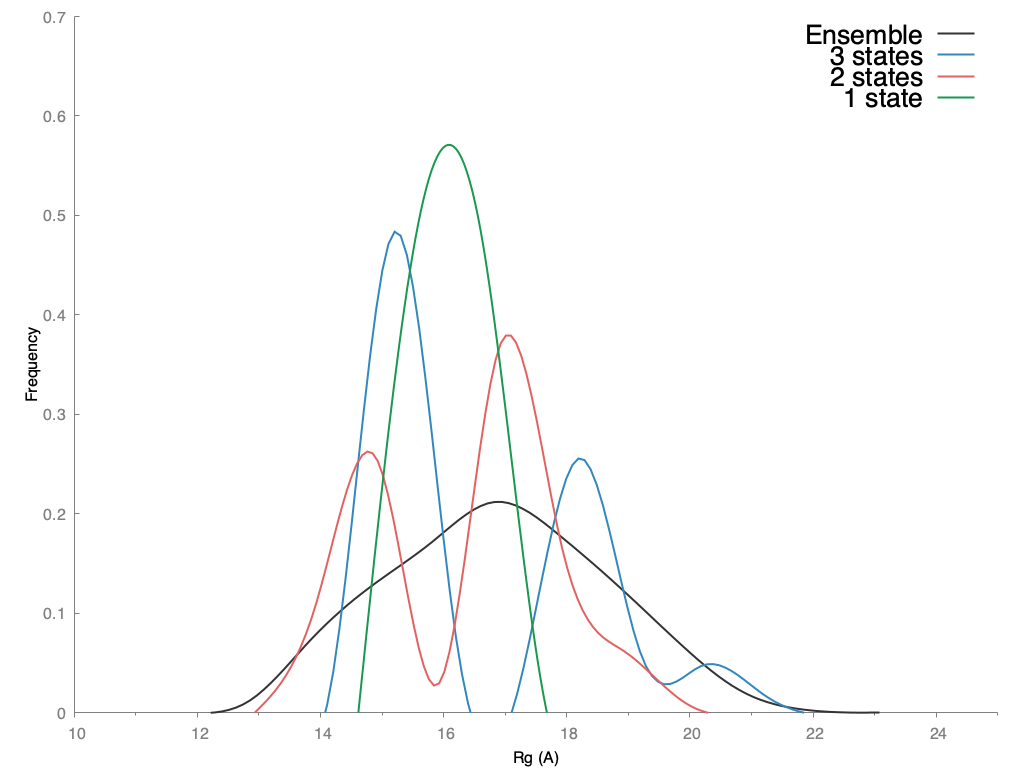 | 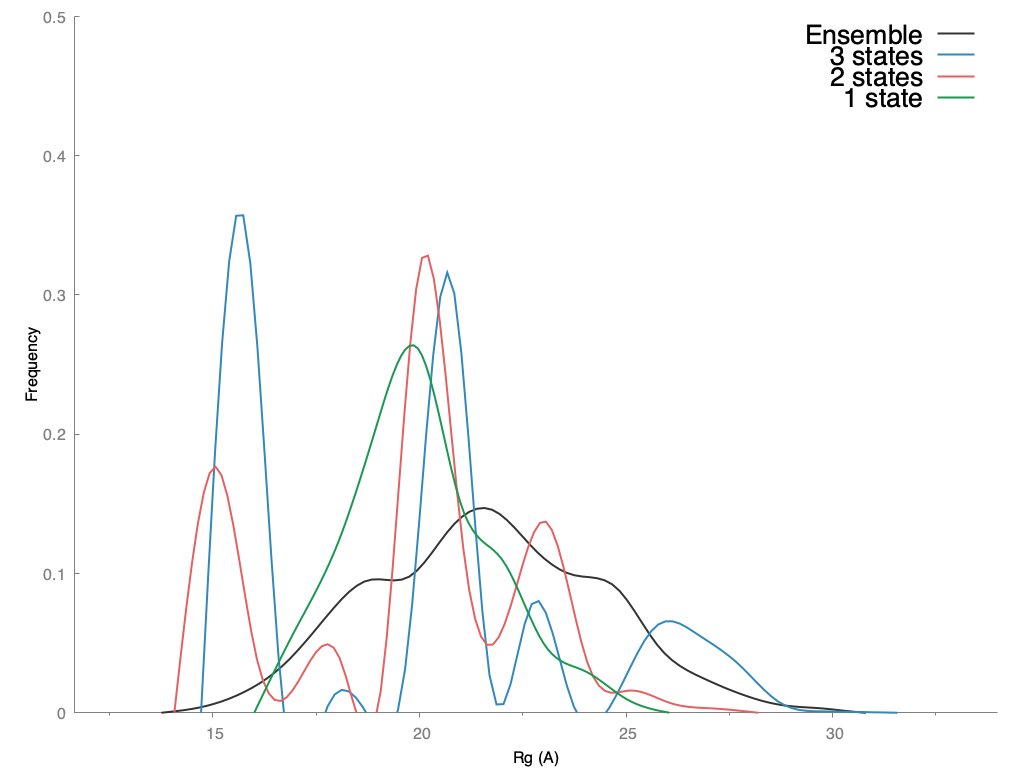 |

**Supplementary Table ST5:** Averaged ^15^N longitudinal (R_1_) and transverse (R_2_) relaxation rates as well as averaged steady-state ^15^N{^1^H} NOE values measured for the different constructs of UIM-SH3 and ubiquitin at 15 °C and at a ^1^H frequency of 600 MHz. Correlation times (τ_c_) have been calculated using the ROTDIF program^4,5^.

|  |  | **R_1_ (s^-1^)** | **R_2_ (s^-1^)** | **NOE** | **τ_c_ (ns)** |
| --- | --- | --- | --- | --- | --- |
| **US-WT** | UIM | 1.65 ± 0.02 | 5.7 ± 0.1 | 0.29 ± 0.01 | 4.40 ± 0.43 |
|  | SH3 | 1.47 ± 0.02 | 10.2 ± 0.2 | 0.74 ± 0.04 | 7.88 ± 0.05 |
| **US-Δ1** | UIM | 1.65 ± 0.04 | 6.7 ± 0.2 | 0.32 ± 0.02 | 4.53 ± 0.21 |
|  | SH3 | 1.45 ± 0.09 | 10.9 ± 0.5 | 0.73 ± 0.04 | 8.10 ± 0.08 |
| **US-Δ2** | UIM | 1.60 ± 0.03 | 6.3 ± 0.1 | 0.33 ± 0.02 | 3.88 ± 0.31 |
|  | SH3 | 1.48 ± 0.03 | 9.9 ± 0.1 | 0.69 ± 0.03 | 7.68 ± 0.07 |
| **US-Δ3** |  | 1.66 ± 0.04 | 8.7 ± 0.4 | 0.76 ± 0.04 | 6.61 ± 0.05 |
| **US-Δ4** |  | 1.60 ± 0.04 | 9.0 ± 0.2 | 0.73 ± 0.03 | 6.90 ± 0.06 |
| **Ub** |  | 1.86 ± 0.01 | 8.0 ± 0.1 | 0.73 ± 0.04 | 5.75 ± 0.01 |

**Supplementary Figure S6:** ^15^N transverse relaxation rate, R_2_, as a function of molecular mass. The respective R_2_ derived for the SH3 or the UIM domain involved in the different constructs are colored black (US-WT), green (US-Δ1), magenta (US-Δ2), blue (US-Δ3) and dark yellow (US-Δ4). R_2_ has been represented for the UIM and SH3 domains of the different constructs by considering a molecular mass of 4.0 kDa and 7.4 kDa respectively. The calibration plain curve was obtained by considering the experimental R_2_ values of UIM, Ub and Lys63-Ub_2_ distal ubiquitin unit and by using the Stokes-Einstein equation^6,7^. This curve can be used as a “molecular mass ruler” and has been obtained by assuming an isotropic overall tumbling of the proteins, a squared order parameter S^2^ of 0.87 and a local correlation time of 50 ps. The dotted black line represents the effect of the anisotropy on R_2_ after averaging the R_2_ values of an ensemble of 50 randomly distributed vectors with S^2^ = 0.87, τ_C_ = 50 ps and an anisotropy of 0.2 (lower curve) and 2.0 (upper curve). We assumed a protein specific density of 0.76 cm^3^/g, a hydration shell of 3.2 Å, and a solvent viscosity of 0.00114 Pa.s at 288 K. The UIM R_2_ value has been taken from a previous study^7^. Ub_2_ stands for Lys63-Ub_2_ chains.


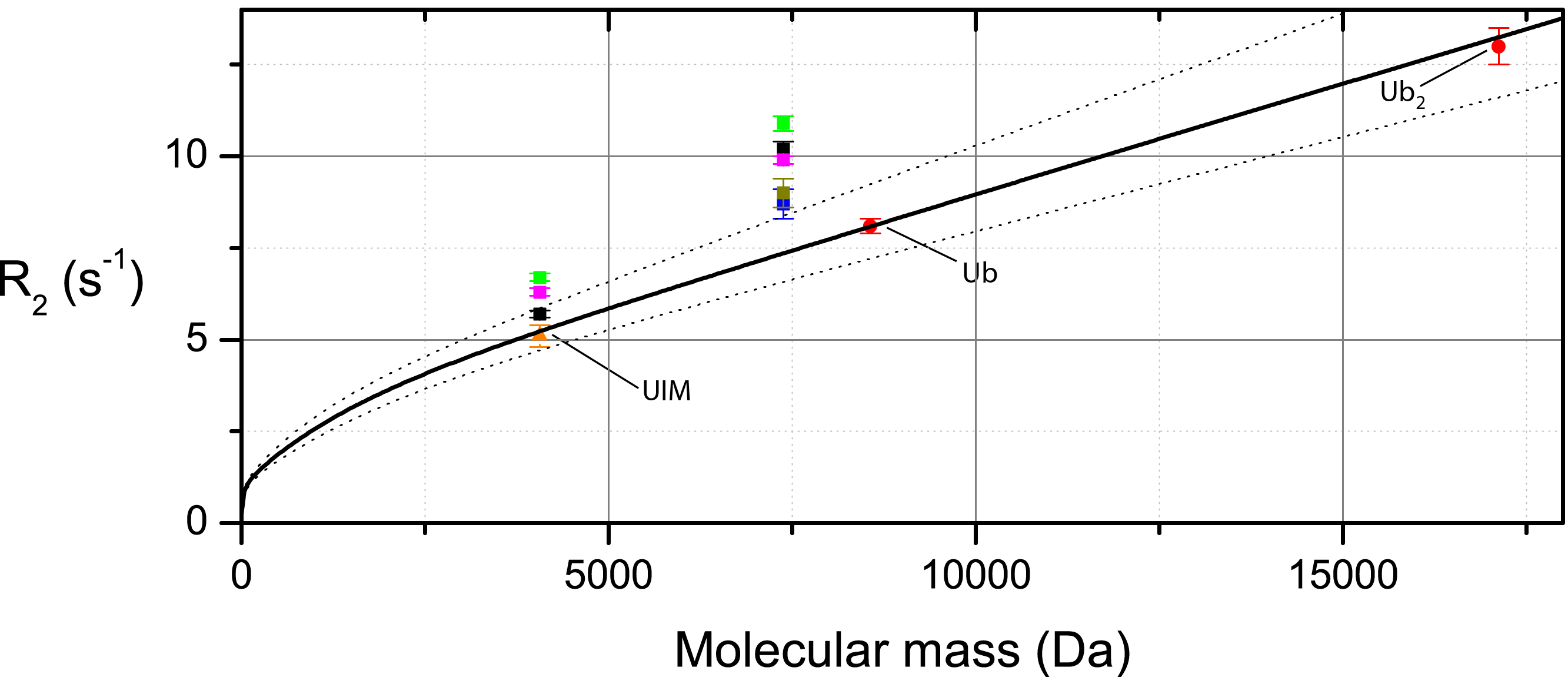


**Reduced spectral density mapping with R_1_, R_2_ and NOE**: Reduced spectral densities were computed at three different frequencies starting from the analytical expression of the three relaxation parameters R_1_, R_2_ and NOE:

$R_{1}=6.25d^{2}J\left( 0.87\omega_{H} \right)+3\left( c^{2}+d^{2} \right)J(\omega_{N})$ (1)

$R_{2}=5.4d^{2}J\left( 0.87\omega_{H} \right)+\left( c^{2}+d^{2} \right)\left[ 2J\left( 0 \right)+\frac{3}{2}J\left( \omega_{N} \right) \right]+R_{ex}$ (2)

$\left( 1-NOE \right)R_{1}\left| \frac{\gamma_{N}}{\gamma_{H}} \right|=5d^{2}J(0.87\omega_{H})$ (3)

where $d=-\left( \frac{\mu_{0}}{4\pi} \right)\left( \frac{\gamma_{H}\gamma_{N}h}{4\pi r_{HN}^{3}} \right)$ and $c=\frac{\gamma_{N}B_{0}\Delta\sigma}{3}$, r_HN_ is the internuclear ^15^N-^1^H distance, Δσ is the anisotropy of the ^15^N chemical shift tensor (CSA), γ_H_, γ_N_, ω_H_, ω_N_ are the gyromagnetic ratios and resonance frequencies of the nuclei, h is Planck's constant, and R_ex_ is the conformational exchange contribution, if any, to measured R_2_. These equations were obtained by modification of the standard expression^8^, assuming that $J\left( \epsilon\omega_{H} \right)=\left( \frac{0.87}{\varepsilon} \right)^{2}J(0.87\omega_{H})$ ^9^. This high frequency component, J(0.87ω_H_) of the spectral density function can be directly determined from R_1_ and NOE, using eq (3):

$$J\left( 0.87\omega_{H} \right)=\frac{\left( 1-NOE \right)}{5d^{2}}R_{1}\left| \frac{\gamma_{N}}{\gamma_{H}} \right|$$

if R_ex_ is negligible,

$$J\left( \omega_{N} \right)=\frac{R_{1}\left\{ 1-1.25(1-NOE)\left| \frac{\gamma_{N}}{\gamma_{H}} \right| \right\}}{3(c^{2}+d^{2})}$$

and

$$J\left( 0 \right)=\frac{R_{2}-R_{1}\left\{ \left| \frac{\gamma_{N}}{\gamma_{H}} \right|\left( 0.455-0.455 NOE \right)+0.5 \right\}}{2(c^{2}+d^{2})}$$

Starting from the data represented in Figure 3, we can represent the corresponding reduced spectral densities in the figure below by assuming an average CSA of -160ppm:

**Supplementary Figure S7:** Reduced spectral densities for each of the US variants using the equations derived above.


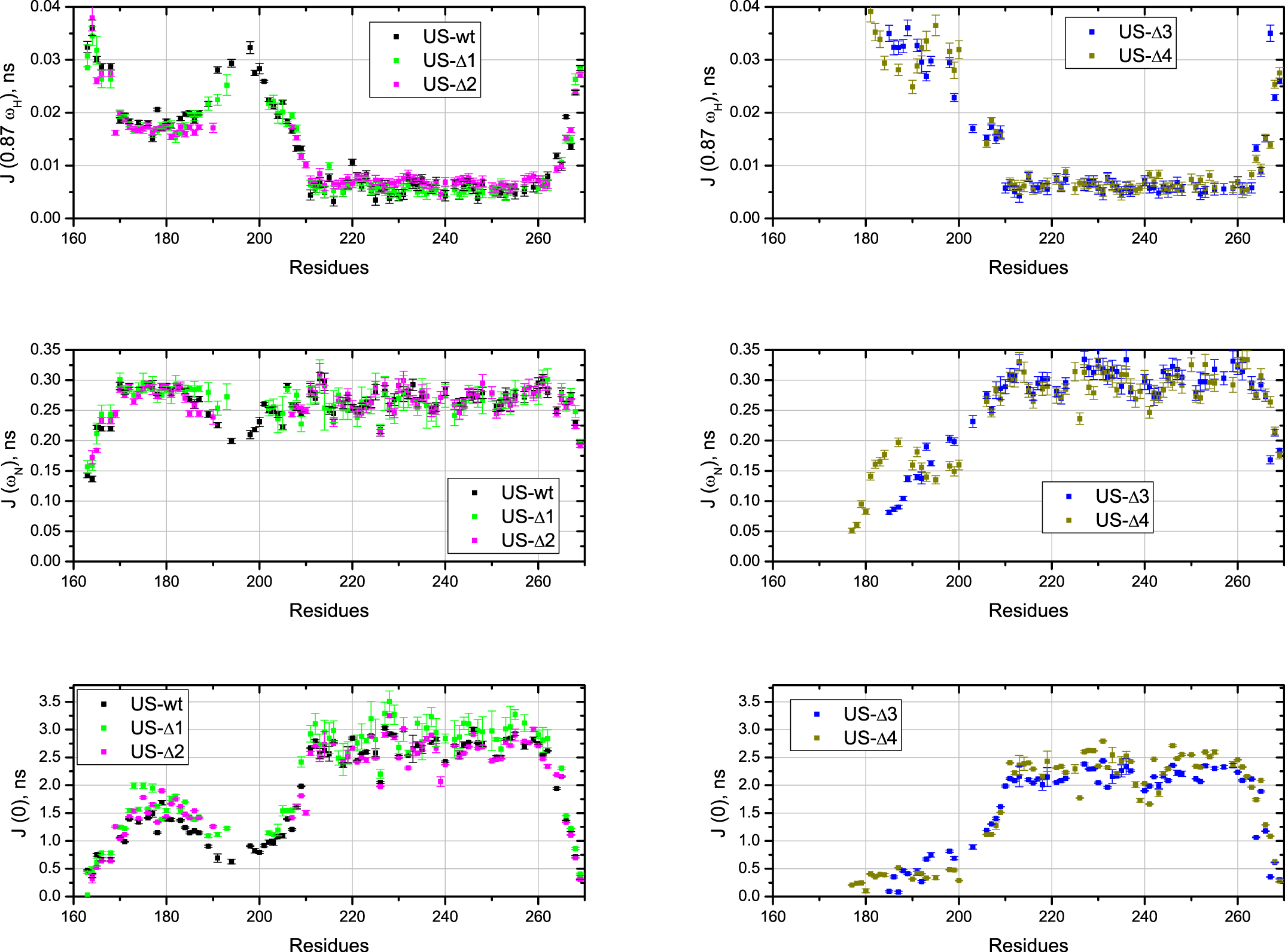


**Supplementary Figure S8:** CSPs observed on ^15^N-labeled (a) US-WT (b) US-Δ1 (c) US-Δ2 (d) US-Δ3 (e) US-Δ4 with unlabeled Ub (left column) and unlabeled Lys63-Ub_2_ (right column).


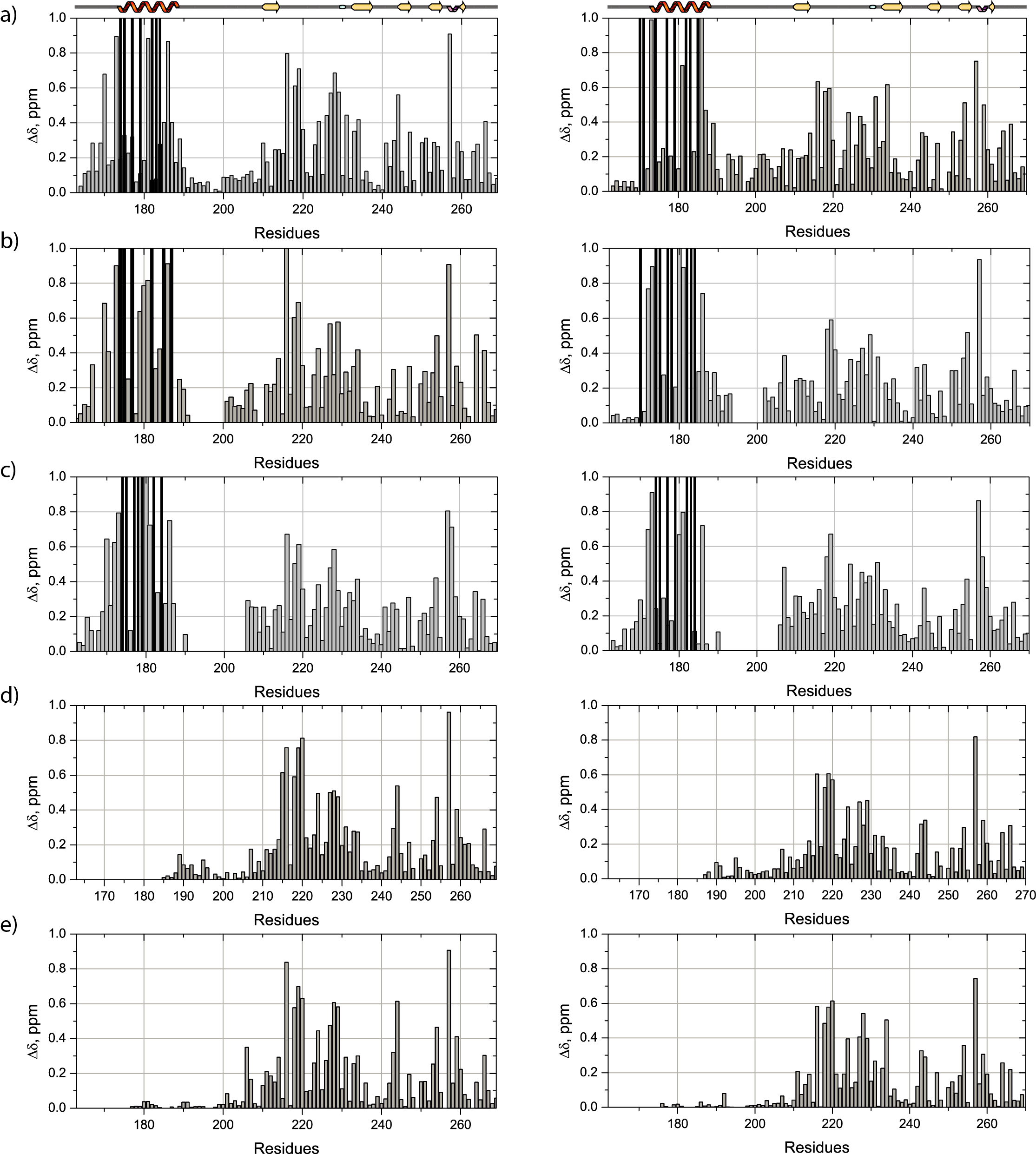


**Supplementary Figure S9:** Titration curves for the different ^15^N-labeled constructs of US with Ub. Only those curves that show final CSPs above a threshold of 0.4 ppm were considered for K_d_ analysis.

**
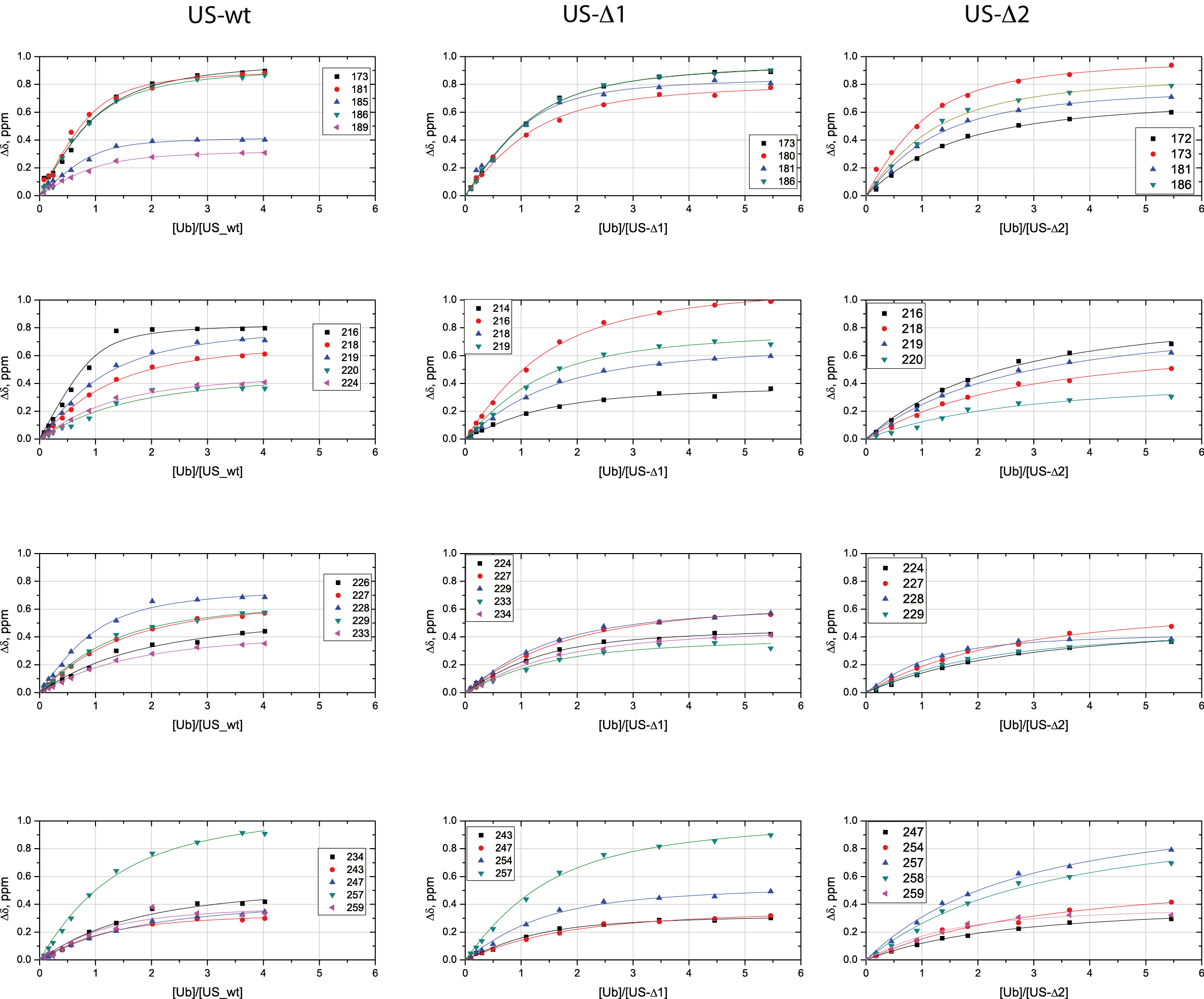
**

**
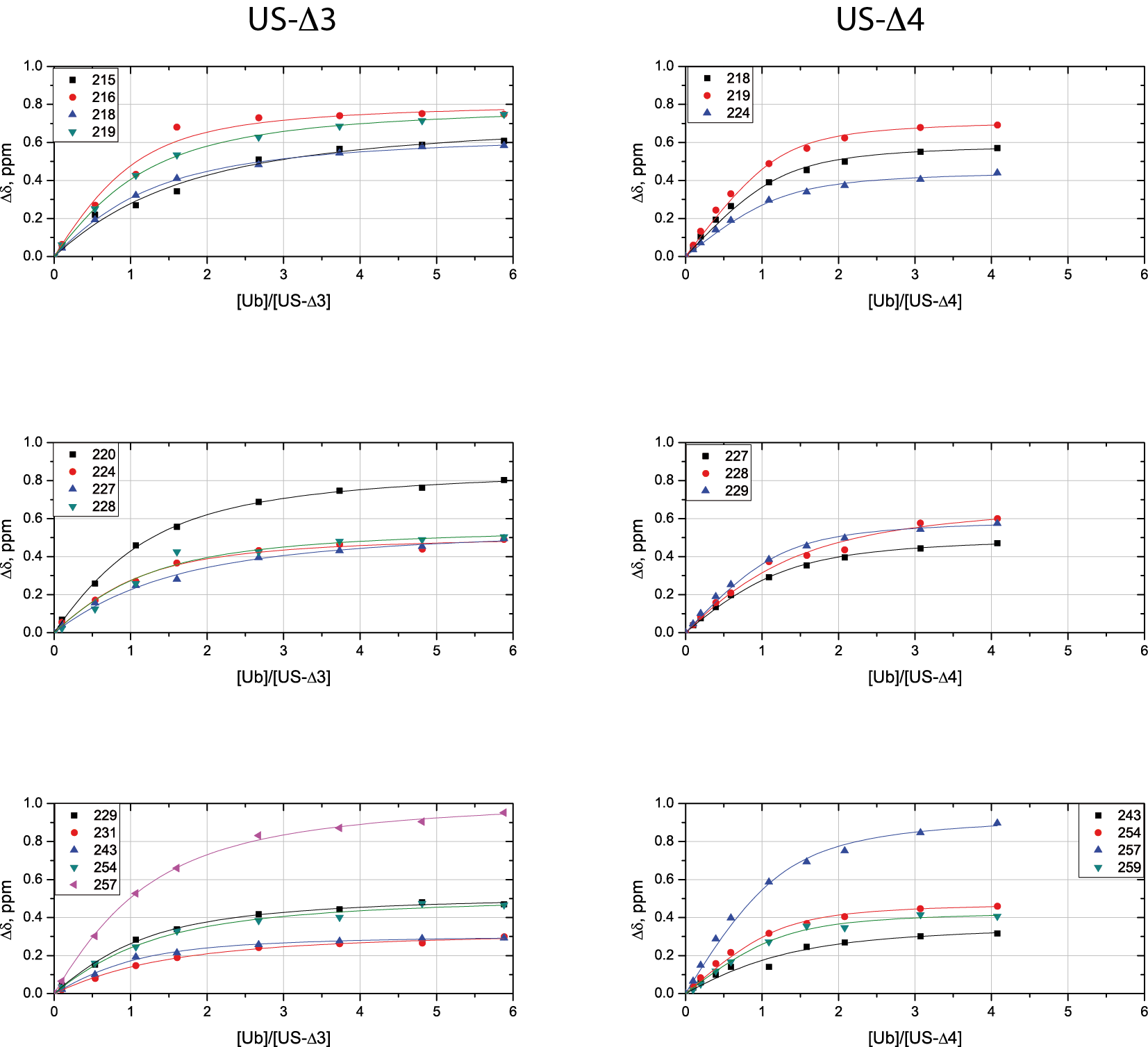
**

**Supplementary Figure S10:** Titration curves for the different ^15^N-labeled US constructs with Lys63-Ub_2_. Only those curves that show final CSPs above a threshold of 0.4 ppm K_d_ were considered for analysis.


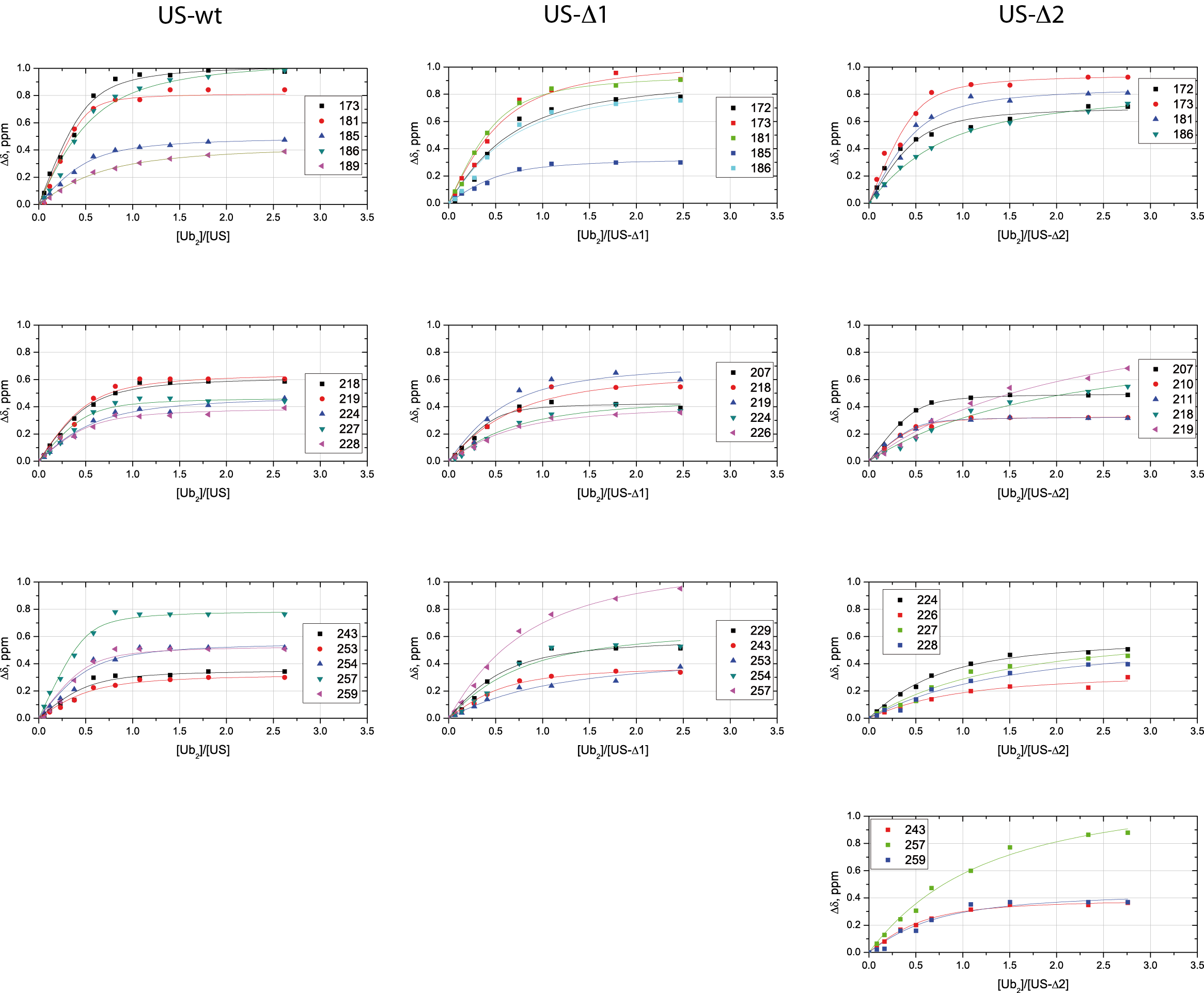

**Supplementary Figure S11:** Effect of the excluded volume on US-WT (left) and US-Δ3 (right) models. To demonstrate that the excluded volume due to UIM (blue) and SH3 (grey) restricts the conformational space available to US-WT compared to US-Δ3, we have added the UIM domain to the N-terminus of US-Δ3 by aligning the UIM C-terminus (His189-Thr192) on the same residues of US-Δ3. This representation clearly shows that the excluded volume due to the UIM and SH3 domains restrict the conformational space available to US-WT while the linker in the US-Δ3 model may adopt a wrap-around conformation and may result in steric clashes if the same orientation of the linker would be used for US-WT (right model). The excluded volume is represented by the solvent accessible surface area and has been calculated using the formula "residue.areaSAS" of Chimera^10^.


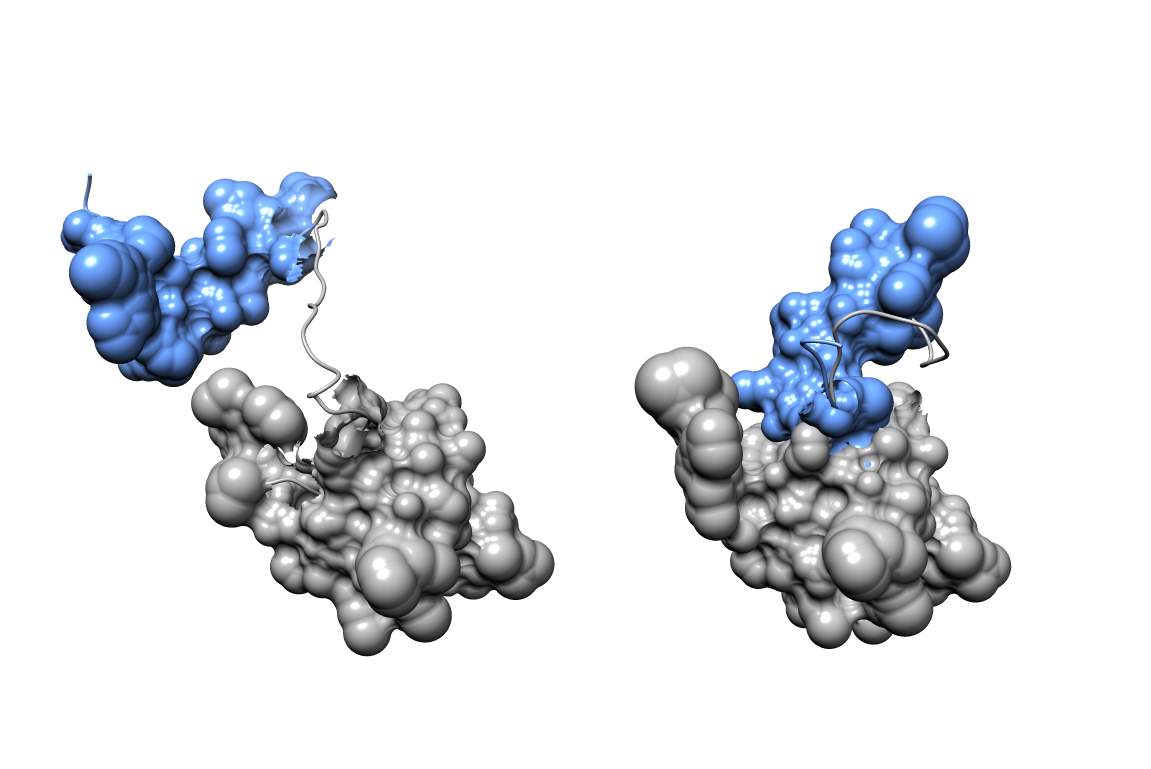


**References**

1. Johnson, W. C. Analyzing protein circular dichroism spectra for accurate secondary structures. *Proteins: Structure, Function, and Bioinformatics* **35**, 307-312, [https://doi.org/10.1002/(SICI)1097-0134(19990515)35:3<307::AID-PROT4>3.0.CO;2-3](https://doi.org/10.1002/(SICI)1097-0134(19990515)35:3%3C307::AID-PROT4%3E3.0.CO;2-3) (1999).

2. Förster, S., Apostol, L. & Bras, W. Scatter: software for the analysis of nano- and mesoscale small-angle scattering. *J Appl Crystallogr* **43**, 639-646, <https://doi.org/10.1107/S0021889810008289> (2010).

3. Schneidman-Duhovny, D., Hammel, M. & Sali, A. FoXS: a web server for rapid computation and fitting of SAXS profiles. *Nucleic Acids Res* **38**, W540-544, <https://doi.org/10.1093/nar/gkq461> (2010).

4. Walker, O., Varadan, R. & Fushman, D. Efficient and accurate determination of the overall rotational diffusion tensor of a molecule from 15N relaxation data using computer program ROTDIF. *J Magn Reson* **168**, 336-345, <https://doi.org/10.1016/j.jmr.2004.03.019> (2004).

5. Berlin, K., Longhini, A., Dayie, T. K. & Fushman, D. Deriving quantitative dynamics information for proteins and RNAs using ROTDIF with a graphical user interface. *J Biomol NMR* **57**, 333-352, <https://doi.org/10.1007/s10858-013-9791-1> (2013).

6. Cavanagh, J., Fairbrother, W. J., Palmer, A. G., Skelton, N. J. & Rance, M. *Protein NMR Spectroscopy*. 2e edition edn, (Academic Press, 2006).

7. Lange, A. *et al.* Evidence for Cooperative and Domain-specific Binding of the Signal Transducing Adaptor Molecule 2 (STAM2) to Lys(63)-linked Diubiquitin. *J Biol Chem* **287**, 18687-18699, <https://doi.org/10.1074/jbc.M111.324954> (2012).

8. Abragam, P. A. & Abragam, A. *The Principles of Nuclear Magnetism*. (Clarendon Press, 1961).

9. Farrow, N. A., Zhang, O., Szabo, A., Torchia, D. A. & Kay, L. E. Spectral density function mapping using 15N relaxation data exclusively. *J Biomol NMR* **6**, 153-162, <https://doi.org/10.1007/BF00211779> (1995).

10. Pettersen, E. F. *et al.* UCSF Chimera—A visualization system for exploratory research and analysis. *Journal of Computational Chemistry* **25**, 1605-1612, <https://doi.org/10.1002/jcc.20084> (2004).
